# In situ tuning of electronic structure of catalysts using controllable hydrogen spillover for enhanced selectivity

**DOI:** 10.1038/s41467-020-18567-6

**Published:** 2020-09-22

**Authors:** Mi Xiong, Zhe Gao, Peng Zhao, Guofu Wang, Wenjun Yan, Shuangfeng Xing, Pengfei Wang, Jingyuan Ma, Zheng Jiang, Xingchen Liu, Jiping Ma, Jie Xu, Yong Qin

**Affiliations:** 1 State Key Laboratory of Coal Conversion, Institute of Coal Chemistry, Chinese Academy of Sciences, 030001 Taiyuan, China; 2 Center of Materials Science and Optoelectronics Engineering, University of Chinese Academy of Sciences, 100049 Beijing, China; 3 Shanghai Synchrotron Radiation Facility, Shanghai Institute of Applied Physics, Chinese Academy of Sciences, 201204 Shanghai, China; 4 State Key Laboratory of Catalysis, Dalian National Laboratory for Clean Energy, Dalian Institute of Chemical Physics, Chinese Academy of Sciences, 116023 Dalian, China

**Keywords:** Catalyst synthesis, Catalytic mechanisms, Heterogeneous catalysis

## Abstract

In situ tuning of the electronic structure of active sites is a long-standing challenge. Herein, we propose a strategy by controlling the hydrogen spillover distance to in situ tune the electronic structure. The strategy is demonstrated to be feasible with the assistance of CoO_*x*_/Al_2_O_3_/Pt catalysts prepared by atomic layer deposition in which CoO_*x*_ and Pt nanoparticles are separated by hollow Al_2_O_3_ nanotubes. The strength of hydrogen spillover from Pt to CoO_*x*_ can be precisely tailored by varying the Al_2_O_3_ thickness. Using CoO_*x*_/Al_2_O_3_ catalyzed styrene epoxidation as an example, the CoO_*x*_/Al_2_O_3_/Pt with 7 nm Al_2_O_3_ layer exhibits greatly enhanced selectivity (from 74.3% to 94.8%) when H_2_ is added. The enhanced selectivity is attributed to the introduction of controllable hydrogen spillover, resulting in the reduction of CoO_*x*_ during the reaction. Our method is also effective for the epoxidation of styrene derivatives. We anticipate this method is a general strategy for other reactions.

## Abstract (web summary)

In situ tuning of the electronic structure of active sites is a long-standing challenge. Here, the authors report an approach to tune the electronic structure of cobalt species during the styrene epoxidation reaction by the introduction of controllable hydrogen spillover for enhanced selectivity.

## Introduction

Highly efficient and selective catalysts are important for the industrial production of commodity chemicals, as well as fine chemicals and pharmaceuticals^1^. The development of new heterogeneous catalysts for carrying out multipath reactions with high selectivity to achieve high-yield production of target chemicals is a longstanding challenge^2,3^. The final product distribution, i.e., the selectivity of a reaction over a given catalyst, is determined by the relative activation barrier among different reaction paths, which in nature, primarily depend on the electronic structure of the catalytic active sites^4–6^. However, under reaction conditions, the electronic structures of active sites are easily affected by temperature, atmosphere, and absorbed species^7,8^. It is difficult to control the active sites to maintain the optimal electronic structure during the reaction, which favors producing more target products. Some researchers have devoted substantial effort to studying how to control and tune the electronic structures of the active sites responsible for activity and selectivity under real reaction conditions^9,10^. In contrast to numerous studies on identifying catalytically active sites under real-time reaction conditions^11–14^, limited methods have been reported for controlling and tuning the electronic structure of active sites during a reaction.

In general, the hydrogen migration from the metal particles to the support, i.e., hydrogen spillover, is a well-known phenomenon in heterogeneous catalysis and is involved in many important reactions^15–24^. The efficiency and spatial extent of hydrogen spillover strongly depend on the types of supports^25,26^. For instance, a well-defined model system demonstrated that on the nonreducible alumina support, hydrogen spillover is limited to short distances, with the hydrogen flux decreasing over distance to create a concentration gradient^25^. In-depth understanding of hydrogen spillover can not only help to explain experimental phenomena but also aid the design of advanced catalysts with enhanced catalytic performances^27–35^. Being inspired by these researches, we posit that there is an opportunity for an approach to modulating the electronic structure of active sites through regulating hydrogen spillover strength for enhanced catalytic performance.

In this work, using the CoO_*x*_ catalyzed epoxidation reaction of styrene as an example, we propose an approach to tune the electronic structure of cobalt species during the reaction by the introduction of controllable hydrogen spillover, to enhance the selectivity of styrene oxide (SO). To demonstrate the strategy, a CoO_*x*_/Al_2_O_3_/Pt catalyst is prepared by a facile and general template-assisted atomic layer deposition (ALD) method^36–38^, in which CoO_*x*_ and Pt nanoparticles are attached on the outer and inner surfaces of Al_2_O_3_ nanotubes, respectively. The strength of hydrogen spillover from Pt to CoO_*x*_ can be precisely tailored by varying the thickness of the Al_2_O_3_ layer. The CoO_*x*_/Al_2_O_3_/Pt catalyst with 7 nm-thick Al_2_O_3_ layer exhibits greatly enhanced selectivity (from 74.3% to 94.8%) when H_2_ atmosphere is introduced. Detailed analyses reveal that the cobalt species under the oxidation condition are reduced to a lower oxidation state by introducing the controllable hydrogen spillover, leading to a higher SO selectivity.

## Results

### Synthesis and characterization of the catalysts

CoO_*x*_/*y*Al_2_O_3_/Pt catalysts with a separating Al_2_O_3_ layer were obtained using carbon nanocoils (CNCs) as sacrificial templates (Supplementary Fig. 1). First, Pt nanoparticles and an Al_2_O_3_ layer were deposited onto CNCs by Pt ALD and Al_2_O_3_ ALD, respectively. Subsequently, the CNC templates were removed by calcination under ambient atmosphere. Finally, CoO_*x*_ nanoparticles were deposited by CoO_*x*_ ALD, producing CoO_*x*_/*y*Al_2_O_3_/Pt (where *y* is the cycle numbers of ALD Al_2_O_3_). For comparison, two reference catalysts (CoO_*x*_/50Al_2_O_3_ and 50Al_2_O_3_/Pt) were also synthesized by a similar method.

**Fig. 1:**
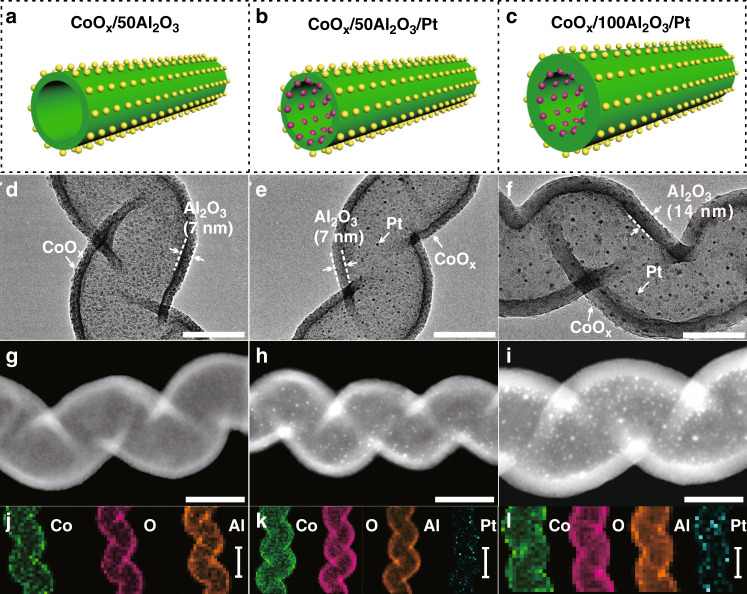
Structural characterization of the catalysts. **a**–**c** Structure diagrams (The green tubes represent Al_2_O_3_. The magenta and yellow balls represent Pt and CoO_*x*_, respectively), **d**–**f** TEM images (scale bar, 50 nm), **g**–**i** HAADF-STEM images (scale bar, 50 nm), and **j**–**l** EDX elemental mappings (scale bar, 100 nm) of the catalysts. **a**, **d**, **g**, **j**: CoO_*x*_/50Al_2_O_3_; **b**, **e**, **h**, **k**: CoO_*x*_/50Al_2_O_3_/Pt; and **c**, **f**, **i**, **l** CoO_*x*_/100Al_2_O_3_/Pt.

**Fig. 2:**
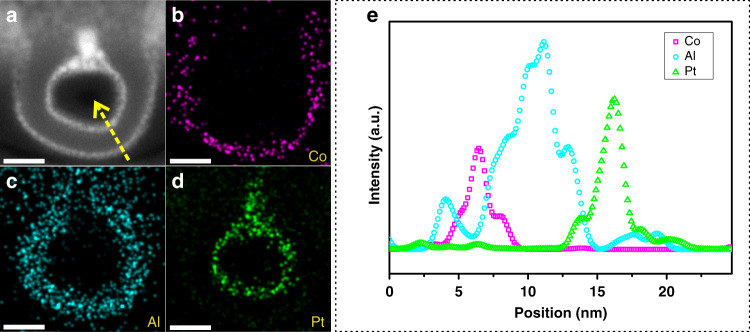
Structural characterization of CoO_*x*_/50Al_2_O_3_/Pt. **a** STEM image (scale bar, 20 nm) and **b**–**d** EDX elemental mappings (scale bar, 20 nm) of a cross-sectional specimen of CoO_*x*_/50Al_2_O_3_/Pt prepared by focused ion beam milling. **e** Compositional point profile of Co, Al, and Pt from the specimen recorded along the yellow arrow shown in **a**.

Figure 1a–c present the structure diagrams of CoO_*x*_/50Al_2_O_3_, CoO_*x*_/50Al_2_O_3_/Pt, and CoO_*x*_/100Al_2_O_3_/Pt. Figure 1d–f show transmission electron microscopy (TEM) images of the three catalysts. TEM images of other catalysts, including CoO_*x*_/*y*Al_2_O_3_/Pt with different cycles of ALD Al_2_O_3_ (35, 65, 80, and 300) and the reference catalyst (50Al_2_O_3_/Pt), are shown in Supplementary Fig. 2. For all TEM images, Al_2_O_3_ hollow structures can be clearly observed. The CoO_*x*_/*y*Al_2_O_3_/Pt with different Al_2_O_3_ cycles (35, 50, 65, 80,100, and 300) possess varied Al_2_O_3_ thicknesses from 5, 7, 9, 11, 14, to 41 nm. Regardless of Al_2_O_3_ thicknesses, the outer CoO_*x*_ and the inner Pt nanoparticles have similar average diameters (Supplementary Figs. 3 and 4), which is consistent with X-ray diffraction (XRD) result (Supplementary Fig. 7 and Supplementary Table 1). A high-resolution TEM (HRTEM) image of CoO_*x*_/50Al_2_O_3_/Pt is shown in Supplementary Fig. 5. The high-angle annular dark field scanning transmission electron microscopy (HAADF-STEM) images (Fig. 1h, i) of CoO_*x*_/50Al_2_O_3_/Pt and CoO_*x*_/100Al_2_O_3_/Pt clearly show that Pt nanoparticles are confined in Al_2_O_3_ nanotubes. Energy-dispersive X-ray spectroscopy (EDS) mappings of the catalysts show that the distributions of Co, O, Al, and Pt (Fig. 1j and l) are consistent with the positions of the CoO_*x*_, Al_2_O_3_, and Pt layers. Further, the distributions of Co, Al, and Pt in CoO_*x*_/50Al_2_O_3_/Pt were revealed clearly by STEM image and EDX mapping (Fig. 2a–d) of a cross-sectional specimen, prepared by focused ion beam (FIB) milling along the vertical direction of the Al_2_O_3_ nanotubes. The line-scanning profile (Fig. 2e) of cross-sectional composition shows that the signal of Co species was not detected in the Al_2_O_3_ nanotubes, which clearly demonstrates the separated structure of CoO_*x*_/50Al_2_O_3_/Pt. The Al peak at ~4.5 nm is ascribed to signal of powder (from the FIB ion milling) remained in the space nearby the outer surface of the sample (Supplementary Fig. 6). The Co and Pt contents in the catalysts, measured by inductively coupled plasma-atomic emission spectrometry (ICP-AES), are shown in Supplementary Table 2. N_2_ sorption experiments (Supplementary Fig. 8 and Supplementary Table 3) demonstrate that all the catalysts possess similar average pore diameter, while their Brunauer–Emmett–Teller (BET) surface areas and pore volumes increase with the decrease of Al_2_O_3_ thicknesses.

**Fig. 3:**
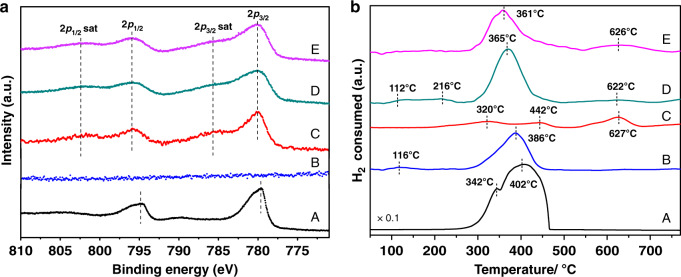
Electronic structure and chemisorption characterizations. **a** XPS Co 2*p* analysis and **b** H_2_-TPR profiles of (A) Co_3_O_4_ as reference, (B) 50Al_2_O_3_/Pt, (C) CoO_*x*_/50Al_2_O_3_, (D) CoO_*x*_/50Al_2_O_3_/Pt, and (E) CoO_*x*_/100Al_2_O_3_/Pt.

X-ray photoelectron spectroscopy (XPS) results (Fig. 3a) reveal the coexistence of Co^3+^ and Co^2+^. Compared to the spinel Co_3_O_4_, the main 2*p* peaks of CoO_*x*_/50Al_2_O_3_, CoO_*x*_/50Al_2_O_3_/Pt, and CoO_*x*_/100Al_2_O_3_/Pt shift to higher binding energy (the 2*p*_3/2_ peaks shift from 779.5 to 780.0 eV and the 2*p*_1/2_ peaks shift from 794.7 to 795.9 eV), and the satellite peaks appear (785.6 eV (2*p*_3/2_ sat) and 802.3 eV (2*p*_3/2_ sat)), indicating that the as-prepared CoO_*x*_ nanoparticles consist of both Co^2+^ and Co^3+^ species^39,40^. Hydrogen temperature-programmed reduction (H_2_-TPR) was also employed (Fig. 3b). Compared to CoO_*x*_/50Al_2_O_3_, the peak intensity of CoO_*x*_/50Al_2_O_3_/Pt centered at 622 °C decreases significantly, which is because the CoO_*x*_ species can be additionally reduced by the spilled active hydrogen from Pt nanoparticles. The peak intensity of CoO_*x*_/100Al_2_O_3_/Pt centered at 626 °C is remained, possibly because the Al_2_O_3_ layer of 100 ALD cycles (14 nm) is too thick and the hydrogen spillover effect is greatly weakened. The differences in reducibility of Co (Supplementary Table 4) demonstrate that the flux of spilled hydrogen species on the nonreducible Al_2_O_3_ support decreases with increasing distance.

### Catalytic performance

The epoxidation of olefins is an important chemical reaction since epoxides are key intermediates in organic synthesis^41,42^. The catalytic performances of different catalysts for the styrene epoxidation reaction with tert-butyl hydroperoxide (TBHP) as the oxidant are summarized in Table 1. Styrene was negligibly converted without a catalyst (Table 1, entry 1). For 50Al_2_O_3_/Pt, only a 17.7% conversion was obtained (Table 1, entry 2). When Co-based catalysts were added, SO was formed as the major product, and benzaldehyde (BzH) was produced as a byproduct. For CoO_*x*_/50Al_2_O_3_, the reaction can be efficiently catalyzed, with 93% conversion and 74.3% SO selectivity (Table 1, entry 3). All of the attempts, including the pre-reduction treatment (Table 1, entry 4), the increase of the ratio of TBHP/styrene (Supplementary Fig. 9), the addition of a Pt component to the catalyst (namely CoO_*x*_/50Al_2_O_3_/Pt) (Table 1, entry 6), and the introduction of H_2_ into the reaction system (H_2_-TBHP) (Table 1, entry 5), failed to enhance the SO selectivity of CoO_*x*_/50Al_2_O_3_. However, when H_2_ was introduced into the reaction, the SO selectivity (94.8%) of CoO_*x*_/50Al_2_O_3_/Pt in the H_2_-TBHP condition (Table 1, entry 7) was remarkably increased by 20.5% compared to CoO_*x*_/50Al_2_O_3_ in the TBHP condition. Although its conversion (82.5%) was slightly reduced, the decreased conversion was easily compensated by prolonging the reaction time. During the entire reaction process, the SO selectivity of the CoO_*x*_/50Al_2_O_3_/Pt in the H_2_-TBHP condition is greatly higher compared with that of the CoO_*x*_/50Al_2_O_3_ in the TBHP condition under the same styrene conversion (Supplementary Fig. 10). After reaction, the distributions of the inner Pt and the outer CoO_*x*_ nanoparticles for CoO_*x*_/50Al_2_O_3_/Pt remain (Supplementary Fig. 11), and no obvious detachment of nanoparticles is observed, indicating that the catalyst is stable during the reaction.

**Table 1:** Catalytic performances of the catalysts for styrene epoxidation reaction in different conditions^a^.

Entry	Atmosphere	Catalysts	Conversion (%)	SO sel. (%)
1	–	–	9.9	37.6
2	–	50Al_2_O_3_/Pt	17.7	72.4
3	–	CoO_*x*_/50Al_2_O_3_	93.0	74.3
4	–	Pre-reduced CoO_*x*_/50Al_2_O_3_^b^	91.2	73.7
5	H_2_	CoO_*x*_/50Al_2_O_3_	89.8	75.9
6	–	CoO_*x*_/50Al_2_O_3_/Pt	93.4	76.2
7	H_2_	CoO_*x*_/50Al_2_O_3_/Pt	82.5	94.8

^a^Reaction conditions: 3.5 mmol styrene, 7 mmol TBHP (70% in water), 20 ml acetonitrile and catalysts with the same CoO_*x*_ content at 80 °C for 8 h.

^b^Reducing CoO_*x*_/50Al_2_O_3_ at 350 °C for 1 h before reaction.

**Fig. 4:**
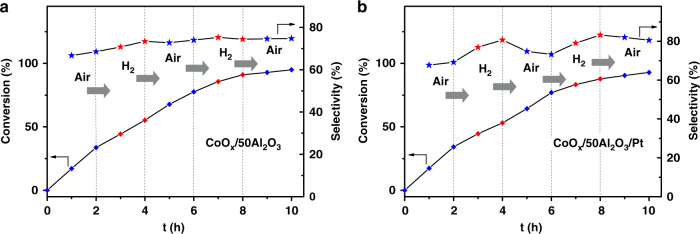
H_2_ pulse experiments. The evolution of styrene conversion and SO selectivity with reaction atmosphere and time over **a** CoO_*x*_/50Al_2_O_3_ and **b** CoO_*x*_/50Al_2_O_3_/Pt.

Further, H_2_ pulse experiments were carried out for CoO_*x*_/50Al_2_O_3_ and CoO_*x*_/50Al_2_O_3_/Pt with alternating pulse of H_2_ and air (Fig. 4a, b). During the reaction, for CoO_*x*_/50Al_2_O_3_, the conversion and selectivity were almost unaffected by the alternate reaction atmosphere. For CoO_*x*_/50Al_2_O_3_/Pt, when air was displaced by H_2_, the increase of conversion slowed down and the SO selectivity was increased obviously. When H_2_ was displaced by air, the opposite phenomenon occurred. This control experiment straightforwardly indicates that the introduction of active hydrogen (Pt and H_2_) plays an essential role in enhancing the selectivity of the catalysts for styrene epoxidation reaction.

The effect of the distance between CoO_*x*_ and Pt components corresponding to the thicknesses of the Al_2_O_3_ layer on the catalytic performance has been investigated (Table 2 and Supplementary Table 5). The thicknesses of the Al_2_O_3_ layer are precisely regulated by varying the cycle numbers of ALD Al_2_O_3_. In TBHP condition, the catalytic performances of CoO_*x*_/*y*Al_2_O_3_/Pt are similar. However, when H_2_ was introduced, their catalytic performances exhibited obvious differences. Varying the cycle numbers of ALD Al_2_O_3_ from 35 to 65, the activities of the three catalysts in the H_2_-TBHP condition decreased compared with those of the corresponding catalysts in the TBHP condition, whereas the SO selectivities show obvious improvements with the highest value of 94.8% for CoO_*x*_/50Al_2_O_3_/Pt (Table 2, entries 1–3). With a further increase of cycle numbers (over 80), the activities and SO selectivities of CoO_*x*_/80Al_2_O_3_/Pt, CoO_*x*_/100Al_2_O_3_/Pt, and CoO_*x*_/300Al_2_O_3_/Pt in the H_2_-TBHP condition are similar to that of the corresponding catalysts in the TBHP condition (Table 2, entries 4–6). At low conversions, CoO_*x*_/50Al_2_O_3_/Pt catalyst also exhibits the greatest improvement of SO selectivity among these catalysts (Supplementary Fig. 12). Therefore, it can be seen that a precisely controlled CoO_*x*_–Pt distance is critically important for the remarkably enhanced SO selectivity.

**Table 2:** Catalytic performances of the catalysts with different Al_2_O_3_ cycles for styrene epoxidation reaction.

Entry	Catalysts	Distance (nm)	TBHP condition		H_2_-TBHP condition	
			Conversion (%)	SO sel. (%)	Conversion (%)	SO sel. (%)
1	CoO_*x*_/35Al_2_O_3_/Pt	5	91.0	77.5	83.5	93.9
2	CoO_*x*_/50Al_2_O_3_/Pt	7	93.4	76.2	82.5	94.8
3	CoO_*x*_/65Al_2_O_3_/Pt	9	92.5	78.2	85.6	86.5
4	CoO_*x*_/80Al_2_O_3_/Pt	11	93.0	76.4	89.0	77.1
5	CoO_*x*_/100Al_2_O_3_/Pt	14	92.0	76.3	90.2	76.8
6	CoO_*x*_/300Al_2_O_3_/Pt	41	94.8	74.8	91.9	75.1

### Catalytic mechanism

To uncover the potential mechanism for the enhanced SO selectivity, a series of experiments and characterizations were carried out. Isotope-labeling experiments were conducted to track the transfer pathways of split hydrogen species. For CoO_*x*_/50Al_2_O_3_/Pt in the D_2_-TBHP condition, the deuterium signal was not found in the mass spectrometry results of BzH and SO (Supplementary Fig. 13), demonstrating that the active hydrogen species were not directly involved in the oxidation of organic substrates.

**Fig. 5:**
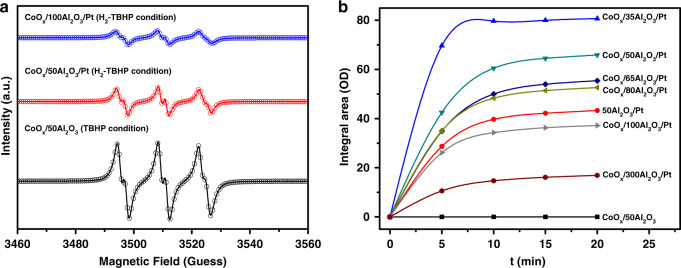
Catalytic mechanism analyses. **a** Experimental EPR spectra (lines) and simulated spectra (open circles) for a mixture of PBN–OOC(CH_3_)_3_ and PBN–OC(CH_3_)_3_. **b** Intensity of the ʋ(OD) band during exposure of samples in (D_2_:H_2_ = 1:1) at a total D_2_/H_2_ flow rate of 30 ml min^−1^ at 80 °C and 1 atm.

A free radical scavenger (butylated hydroxytoluene, BHT) was added to the reaction to further study the reaction mechanism. For CoO_*x*_/50Al_2_O_3_ in the TBHP condition, and CoO_*x*_/50Al_2_O_3_/Pt and CoO_*x*_/100Al_2_O_3_/Pt in the H_2_-TBHP condition, the conversion of styrene stopped after the addition of BHT (Supplementary Fig. 14), indicating that the radical pathway has an important contribution to the reaction mechanism. Moreover, according to the electron paramagnetic resonance (EPR) results (Fig. 5a, Supplementary Fig. 15, and Supplementary Table 6), two kinds of radicals, i.e., tert-butylperoxy and tert-butyloxy radicals (tBuOO∙ and tBuO∙), can be detected in the three cases. These results indicate that the styrene epoxidation reaction over Co-based catalysts experienced a radical process, and the kinds of the radicals were the same in the three cases.

The existence of hydrogen spillover from Pt to CoO_*x*_ through the nonreducible Al_2_O_3_ support was confirmed by H–D exchange experiments (Fig. 5b and Supplementary Fig. 16). Figure 5b shows the H–D exchange rates of CoO_*x*_/50Al_2_O_3_, 50Al_2_O_3_/Pt, and CoO_*x*_/*y*Al_2_O_3_/Pt (*y* = 35, 50, 65, 80, 100, and 300), indicating that the flux of spilled deuterium species from Pt to CoO_*x*_ on the nonreducible Al_2_O_3_ support decreases with increasing distance. The hydrogen spillover was also confirmed by the color change in the mixture of the catalyst and WO_3_ nanowires (Supplementary Fig. 17). There are debates on the existence of hydrogen spillover on nonreducible support (SiO_2_, Al_2_O_3_, and zeolite). It was argued that hydrogen spillover to defect-free surfaces of nonreducible metal oxides cannot take place, but spillover to a non-reducible support with defects is possible^16^. In recent years, more and more evidence demonstrates that although Al_2_O_3_ is a non-reducible oxide, hydrogen spillover can occur on it^25,43–49^. In this work, the obtained Al_2_O_3_ is amorphous (Supplementary Fig. 7) and thus many defects exist. The nonreducible Al_2_O_3_ support was calcined at high temperature, which may result in the formation of a small amount of micropores^50^. Thus, the active hydrogen species may spill over the surface of the micropores or through the defects of Al_2_O_3_ layer.

**Fig. 6:**
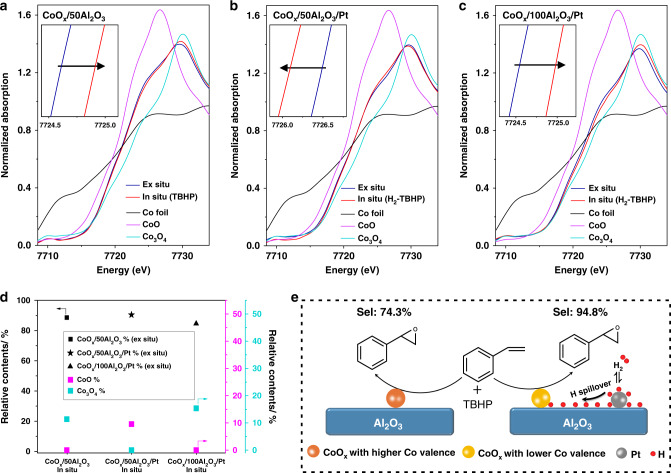
XANES spectra of the catalysts and proposed mechanisms. **a**–**c** Ex situ and in situ Co K-edge XANES spectra of CoO_*x*_/50Al_2_O_3_, CoO_*x*_/50Al_2_O_3_/Pt, and CoO_*x*_/100Al_2_O_3_/Pt, and spectra of reference samples (Co foil, CoO, and Co_3_O_4_). Insets show the expanded sections of absorption edges. **d** The results of the linear combination fitting for the in situ spectra of the catalysts. For each catalyst, the in-situ spectrum is fitted by a linear combination of the ex-situ spectrum and the spectra of reference samples (Co_3_O_4_ and CoO). **e** Proposed reaction mechanisms for CoO_*x*_/50Al_2_O_3_ in the TBHP condition and CoO_*x*_/50Al_2_O_3_/Pt in the H_2_-TBHP condition. In the presence of controllable hydrogen spillover, cobalt species with a lower valence state are beneficial to the enhanced selectivity.

To reveal the electronic structures of cobalt species, X-ray absorption fine structure (XAFS) of the catalysts was investigated (Fig. 6a–c). The ex situ X-ray absorption near-edge structure (XANES) profiles show that the positions of the white line peaks for the CoO_*x*_/50Al_2_O_3_, CoO_*x*_/50Al_2_O_3_/Pt, and CoO_*x*_/100Al_2_O_3_/Pt are all located between the peaks of the rock-salt CoO and spinel Co_3_O_4_, indicating that the oxidation states of cobalt species for as-prepared catalysts include both Co^3+^ and Co^2+^, which is consistent with the XPS result. In the presence of TBHP, compared with the ex situ spectrum, the absorption edge of the in-situ spectrum for CoO_*x*_/50Al_2_O_3_ shifted to higher energy (Fig. 6a), revealing that the cobalt species in the reaction is at a higher oxidation state. The changes in the valence of catalyst are not significant, because of the mild reaction conditions (80 °C and atmosphere pressure). When H_2_ was introduced into the reaction system, no obvious difference was observed in the H_2_-TBHP condition compared with in the TBHP condition for CoO_*x*_/50Al_2_O_3_ (Supplementary Fig. 18). For CoO_*x*_/100Al_2_O_3_/Pt, a distinct increase in the cobalt oxidation state was also observed in the reaction (H_2_-TBHP condition) (Fig. 6c). However, for CoO_*x*_/50Al_2_O_3_/Pt, the absorption edge of the in-situ spectrum shifted to lower energy (Fig. 6b), meaning a decrease in the cobalt oxidation state in the H_2_-TBHP condition. To quantitatively reveal the change in the cobalt oxidation states during the reaction, the in-situ XANES spectrum is simulated by a linear combination of the ex-situ spectrum of the as-prepared catalyst and the spectra of reference samples (Co_3_O_4_ and CoO) (Supplementary Fig. 19 and Supplementary Table 7). For CoO_*x*_/50Al_2_O_3_ in the TBHP condition and CoO_*x*_/100Al_2_O_3_/Pt in the H_2_-TBHP condition, extra Co_3_O_4_ (11.4% and 15.4%, respectively) is formed, as shown in Fig. 6d. However, for CoO_*x*_/50Al_2_O_3_/Pt in the H_2_-TBHP condition, extra CoO (9.6%) is formed. The *k*^2^-weighted Fourier-transformed extended X-ray absorption fine structure (FT-EXAFS) spectra (Supplementary Fig. 20) and their curve fitting results (Supplementary Table 8) are consistent with the conclusions drawn from the XANES experiments.

## Discussion

Our results show that epoxidation selectivity can be obviously enhanced after controllable hydrogen spillover was introduced. The possible reasons of the enhanced selectivity were revealed by detailed experiments and characterizations.

After Pt and H_2_ were introduced into the epoxidation reaction, the CoO_*x*_ catalyzed epoxidation reaction still experienced a radical process and the kinds of radicals were the same, which can be concluded from the radical scavenging and EPR results. On the other hand, for CoO_*x*_/*y*Al_2_O_3_/Pt, they are similar in sizes of nanoparticles (TEM and XRD) and pore diameter (N_2_ sorption) regardless of the thicknesses of Al_2_O_3_. Their catalytic performances are similar in TBHP condition. Moreover, the pre-reduction treatment of CoO_*x*_/50Al_2_O_3_ further indicates the enhanced selectivity is not ascribed to the ex-situ oxidation states of fresh catalysts.

In contrast, when H_2_ was introduced into the reaction, the catalytic performances of the CoO_*x*_/*y*Al_2_O_3_/Pt with different Al_2_O_3_ thicknesses exhibited obvious differences. The color change of WO_3_ nanowires and H–D exchange experiments suggest that when H_2_ was introduced into the reaction system, hydrogen spillover can occur and the strength of hydrogen spillover decreases with increasing distance, which is in good accordance with the TPR result. Moreover, isotope-labeling experiments demonstrate that the active hydrogen species are not directly involved in the oxidation of organic substrates. For the catalyst with enhanced selectivity, the CoO_*x*_–Pt distance (several nanometers) is comparable to the distance of hydrogen spillover on Al_2_O_3_, implying that the controllable hydrogen spillover is responsible for the enhanced selectivity. The in situ XAFS results further confirm the explanation. It clearly reveals that the electronic structures of cobalt species during the epoxidation reaction can be successfully modulated through controlling the hydrogen spillover distance. For CoO_*x*_/50Al_2_O_3_/Pt in H_2_-TBHP condition, the cobalt species are reduced to a lower oxidation state, while for CoO_*x*_/50Al_2_O_3_ in TBHP condition and CoO_*x*_/100Al_2_O_3_/Pt in H_2_-TBHP condition, the cobalt species are oxidized to a higher oxidation state. For the epoxidation reaction, one common understanding of the reaction mechanism is recognized that catalysts affect the reactions by bonding of oxygen to the metal^51–54^. The electronic structure of catalyst at reaction conditions affects the strength of the Co–O bond in the catalyst, which further determines the selectivity of epoxides.

In addition to hydrogen spillover on the Al_2_O_3_ support, other possible migration mechanisms of active hydrogen species, including carbon impurities and water-assisted H shuttling, are also considered. Carbon impurities are proposed as an alternative hydrogen migration path on nonreducible support^16^. Water-assisted H shuttling can accelerate proton transfer and is proposed to explain the enhanced performance observed in the presence of water for hydrogenation reactions^55,56^. However, the distance of hydrogen spillover on carbon species is far beyond the range of several nanometers^11,24^. Similarly, hydrogen shuttling is a remote promotional effect^57^. These mechanisms cannot fit our catalytic results well.

Based on all above results, it can be concluded that the enhanced selectivity is attributed to the introduction of controllable hydrogen spillover, which is further confirmed by the H_2_ pulse experiments. In the presence of controllable hydrogen spillover, cobalt species with a lower valence state are more beneficial for enhanced catalytic selectivity, as shown in Fig. 6e. Based on the successful demonstration of in-situ tailoring of Co valence by hydrogen spillover, we can expect that the selectivity can also be changed over a CoO_*x*_Pt/50Al_2_O_3_ catalyst with the closely contacted Pt and CoO_*x*_ (Supplementary Fig. 21) due to the effect of hydrogen spillover. As expected, the SO selectivity of CoO_*x*_Pt/50Al_2_O_3_ in the H_2_-TBHP condition is also increased compared to that in the TBHP condition (Supplementary Table 9). This further confirms that the introduction of controllable hydrogen spillover is responsible for the enhanced selectivity.

The strategy by introducing controllable hydrogen spillover into reaction was also successfully applied to the epoxidation of a variety of styrene derivatives (Table 3). In all cases, CoO_*x*_/50Al_2_O_3_/Pt exhibits greatly enhanced selectivities (up to over 90%) via the introduction of controllable hydrogen spillover. Further, the Pt/50Al_2_O_3_/CoO_*x*_ (Supplementary Fig. 22), which was produced by exchanging the deposition sequences of Pt and CoO_*x*_ nanoparticles, also exhibits enhanced SO selectivity through the introduction of controllable hydrogen spillover (Supplementary Table 10). In addition to cobalt catalysts, iron catalysts were also applied for styrene epoxidation. The SO selectivity of FeO_*x*_/50Al_2_O_3_/Pt in the H_2_-TBHP condition is higher than that of FeO_*x*_/50Al_2_O_3_ in the TBHP condition (Supplementary Fig. 23). These results demonstrate that the introduction of controllable hydrogen spillover for enhanced selectivity is a general method.

**Table 3:** Catalytic epoxidation of styrene derivatives in different conditions^a^.

Catalysts	Condition	Substrates	*t* (h)	Conversion (%)	Epoxide sel. (%)
CoO_*x*_/50Al_2_O_3_	TBHP	*p*-methylstyrene	12	97.1	88.4
		*p*-methoxy-styrene	12	80.5	78.2
		*m*-nitrostyrene	16	68.4	68.2
		*m*-chlorostyrene	12	88.7	69.9
		2-vinyl naphthalene	12	82.1	79.8
CoO_*x*_/50Al_2_O_3_/Pt	H_2_-TBHP	*p*-methylstyrene	12	86.2	98.2
		*p*-methoxy-styrene	12	68.1	94.3
		*m*-nitrostyrene	16	59.2	92.3
		*m*-chlorostyrene	12	81.1	89.4
		2-vinyl naphthalene	12	71.5	91.5

^a^Reaction conditions: 3.5 mmol substrates, 7 mmol TBHP (70% in water), 20 ml acetonitrile, and catalysts with the same CoO_*x*_ content at 80 °C.

In summary, we have demonstrated a strategy, involving the introduction of controllable hydrogen spillover to the styrene epoxidation reaction to in situ tune the electronic structure of cobalt species for enhanced SO selectivity. The strength of hydrogen spillover from Pt nanoparticles to CoO_*x*_ was tuned by altering the thickness (ALD cycles) of the separating Al_2_O_3_ layer. When the thickness of the Al_2_O_3_ layer was 7 nm, the catalyst (CoO_*x*_/50Al_2_O_3_/Pt) in the H_2_-TBHP condition exhibited significantly enhanced SO selectivity (over 20%) compared with the CoO_*x*_/50Al_2_O_3_ catalyst in the TBHP condition. Our method of in situ electronic structure regulation, achieved through controllable hydrogen spillover, can be used for other catalytic reactions.

## Methods

### Synthesis of 50Al_2_O_3_/Pt catalysts

The synthesis of CNCs and the ALD process can be found in the Supplementary Methods. CNCs were firstly deposited with Pt nanoparticles (20 cycles) and then coated with an Al_2_O_3_ layer (50 cycles) by ALD, producing 50Al_2_O_3_/Pt/CNCs. The 50Al_2_O_3_/Pt/CNCs were calcinated at 500 °C for 1 h in air to remove the CNC templates, obtaining 50Al_2_O_3_/Pt catalysts.

### Synthesis of CoO_*x*_/50Al_2_O_3_ catalysts

CNCs were firstly coated with an Al_2_O_3_ layer (50 cycles) by ALD producing Al_2_O_3_/CNCs, which were calcinated at 500 °C for 1 h in air. Then the obtained hollow Al_2_O_3_ nanotubes were deposited with CoO_*x*_ nanoparticles (35 cycles) by ALD, obtaining CoO_*x*_/50Al_2_O_3_ catalysts.

### Synthesis of CoO_*x*_/*y*Al_2_O_3_/Pt catalysts

CNCs were firstly deposited with Pt nanoparticles and then coated with an Al_2_O_3_ layer (*y* = 35, 50, 65, 80, 100, 300) by ALD. After Al_2_O_3_ deposition, CNCs were completely enclosed by compact ALD Al_2_O_3_. The obtained samples were calcinated at 500 °C for 1 h in air and subsequently coated with CoO_*x*_ nanoparticles (35 cycles) by ALD, obtaining CoO_*x*_/*y*Al_2_O_3_/Pt.

### Synthesis of CoO_*x*_Pt/50Al_2_O_3_ catalysts

The hollow Al_2_O_3_ nanotubes prepared by the above method were deposited with Pt (20 cycles) and CoO_*x*_ nanoparticles (35 cycles) by ALD, obtaining CoO_*x*_Pt/50Al_2_O_3_ catalysts.

### Synthesis of Pt/50Al_2_O_3_/CoO_*x*_ catalysts

CNCs were firstly deposited with CoO_*x*_ nanoparticles (35 cycles) and then coated with an Al_2_O_3_ layer (50 cycles) by ALD. The obtained samples were calcinated at 500 °C for 1 h in air and subsequently coated with Pt nanoparticles (20 cycles) by ALD, obtaining Pt/50Al_2_O_3_/CoO_*x*_.

### Synthesis of FeO_*x*_/50Al_2_O_3_ and FeO_*x*_/50Al_2_O_3_/Pt catalysts

The hollow Al_2_O_3_ nanotubes and the 50Al_2_O_3_/Pt catalysts prepared by the above method were deposited with FeO_*x*_ nanoparticles (70 cycles) by ALD, obtaining FeO_*x*_/50Al_2_O_3_ and FeO_*x*_/50Al_2_O_3_/Pt catalysts, respectively.

### Catalyst characterizations

FIB based on high-brightness Ga liquid-metal ion sources was recorded in FIB-SEM instrument (LYRA3 XMH, TESCAN). Typically, CNCs were first dispersed on a Si substrate and then were deposited by Pt and Al_2_O_3_ ALD. After calcination, the CoO_*x*_ was deposited. Then the Si substrate with the CoO_x_/50Al_2_O_3_/Pt sample was transferred into the FIB-SEM system. The sample was first protected by a carbon layer. After that, a selected part of the protected CoO_*x*_/50Al_2_O_3_/Pt was lifted out of the Si substrate by FIB milling using Ga ion beam and then mounted on a TEM grid. Finally, the sample was sliced down to ~100 nm along the vertical direction of the Al_2_O_3_ nanotubes by the Ga ion beam for TEM analysis. The EPR spectrums of the reaction solutions were recorded on a Bruker EMX spectrometer (EMXplus-10/12) using the following settings: frequency 9.862 GHz, sweep width 3460.0 G, time constant 60 ms, modulation frequency 100 kHz, modulation width 0.5 G, microwave power 5 mW. The spin trap N-tert-butyl-α-phenylnitrone (PBN) was added into the reaction solutions to form radical adducts (1:1 molar ratio between the spin trap and TBHP). The solutions were filtered and the spectra were obtained at room temperature (298 K), using capillary tubes with the same dimensions as those used for the recording of the spectra of the catalysts. H–D exchange was characterized by in-situ infrared (IR) spectroscopy. IR spectra were recorded with a Bruker IFS 66v/S spectrometer with a resolution of 2 cm^−1^, and each spectrum is an average of 64 scans. The sample for IR spectroscopy was loaded into a diffuse reflectance infrared Fourier transform spectroscopy (DRIFTS) cell (Harrick Scientific Products, Praying MantisTM). The cell was connected to a flow system that allows recording of spectra while gases pass through and around the sample. The samples in IR cell were pretreated at 473 K for 1 h and then cooled down to 353 K in flowing He (30 mL min^−1^). After the treatment, the samples were exposed to flowing gas (H_2_ at 30 mL min^−1^) at 353 K for 1 h, and then switched to flowing gas mixtures (H_2_ at 15 mL min^−1^, D_2_ at 15 mL min^−1^) at 353 K. The spectra of H–D exchange were recorded. The ex situ and in situ XAFS were obtained on the 1W1B beamline of the Beijing Synchrotron Radiation Facility (BSRF), Institute of High Energy Physics, Chinese Academy of Sciences, and the BL14W1 beamline of the Shanghai Synchrotron Radiation Facility (SSRF), Shanghai Institute of Applied Physics, Chinese Academy of Sciences. A Si (111) double-crystal monochromator was used to reduce the harmonic component of the monochrome beam. Co foil, CoO, and Co_3_O_4_ were used as reference samples and measured in transmission mode. IFEFFIT software was used to calibrate the energy scale, to correct the background signal and to normalize the intensity. The theoretical paths for Co–O and Co–Co species used for fitting three coordination shells of the experimental data were generated using the FEFF7 program. The coordination number, bond distance, Debye–Waller factor, and inner potential correction were used as variable parameters for the fitting procedures. The in-situ XANES spectrum was simulated by a linear combination of the ex-situ spectrum of the as-prepared catalyst and the spectra of reference samples (Co_3_O_4_ and CoO). The following formula was used: (in situ XANES) = *f*_1_·(ex situ XANES) + *f*_2_·(XANES of Co_3_O_4_) + *f*_3_·(XANES of CoO), where *f*_1_, *f*_2_, and *f*_3_ are the fractions of the as-prepared catalyst, Co_3_O_4_ and CoO, respectively.

The other characterizations are provided in the Supplementary Methods.

### Catalytic activity measurements

Styrene epoxidation reactions over the catalysts were carried out in a 50 ml three-necked round bottom flask equipped with a magnetic stirrer in an oil bath. A mixture of catalysts with the same CoO_*x*_ content, 20 ml acetonitrile, 3.5 mmol styrene, and 7 mmol TBHP (70% TBHP in water) was introduced into the reaction vessel. Then, the reaction mixture was magnetically stirred and heated to 80 °C at atmospheric pressure. When H_2_ was introduced into the reaction, the vessel was purged with purified hydrogen to remove the air at atmospheric pressure, and then was completely sealed with rubber plugs. Heating the reaction system to 80 °C, styrene was injected through needle tube. After reaction for 8 h, the reaction mixture was separated and the liquid products collected were analyzed by gas chromatography–mass spectrometry (GC–MS, Agilent Technologies 7890A-5795C) with a capillary column (HP-5, 30 m × 25 mm × 0.25 μm). Free radical capture experiments were carried out by adding 2 mmol quenchers (BHT) into the reaction system after 1 h reaction. H_2_ pulse experiments were carried out in a 50 ml three-necked round bottom flask with alternating pulse of H_2_ and air. When the reaction atmosphere was displaced, the heating was stopped and each displaced process was maintained for 10 min with the gas flow rate of 30 sccm. Note that before each pulse, air or H_2_ was purged by inert gas nitrogen to ensure the safety. After displacement, the vessel was completely sealed and continued to be heated. The samples were collected every hour, and the reaction atmosphere was displaced every 2 h.

## Safety notices

Mixing hydrogen with an oxidant, in principle, may be not safe. In this paper, the risks are very minor considering mild reaction condition (low reaction temperature, atmospheric pressure, a small amount of H_2_ (30  ml), and a round bottom flask sealed with rubber plugs). If readers attempt to repeat it, please refer to the detailed experiment process in the section of “Catalytic activity measurements”.

## Supplementary information

Supplementary Information

## Data Availability

The data that support the findings of this study are available from the corresponding author under reasonable request.
