# Supplementary material for: In situ tuning of electronic structure of catalysts using controllable hydrogen spillover for enhanced selectivity
Source: Nat Commun. 2020 Sep 22;11:4773. doi: 10.1038/s41467-020-18567-6 (PMC7508871; doi:10.1038/s41467-020-18567-6)
Supplement: Supplementary file 1 — Supplementary Information [file 41467_2020_18567_MOESM1_ESM.pdf]

Supplementary Information

# In situ tuning of electronic structure of catalysts using controllable hydrogen spillover for enhanced selectivity

Xiong et al.

**Supplementary Methods**

**Supplementary Figures 1-23**

**Supplementary Tables 1-10**

**Supplementary Notes**

**Supplementary References**

## Supplementary Methods

**Synthesis of CNCs and the ALD process.** The CNCs were synthesized by chemical vapor deposition using acetylene as a carbon source and copper nanoparticles as catalysts at 250 $^oC$ followed by a heat treatment at 900 $^oC$ in an Ar atmosphere for 2 h. Raw CNCs were refluxed in $HNO_3$ (15 wt %) for 4 h at 100 $^oC$ in an oil bath to remove the copper catalysts, then filtered and washed with deionized water and ethanol until there was no further change in pH (around pH = 7).

The ALD process was carried out in a hot-wall closed chamber-type ALD reactor. Prior to ALD, the CNCs (3 g) were dispersed in ethanol (100 mL) by ultrasonic agitation, and then 1 mL of the suspension was dropped onto a quartz wafer (10 cm×10 cm). After the samples were dried at ambient temperature, they were transferred to the ALD chamber. Pt nanoparticles were deposited at 250 $^oC$ with trimethyl(methylcyclopentadienyl) platinum ($MeCpPtMe_3$) and ozone ($O_3$) as precursors. $MeCpPtMe_3$ was kept at 65 $^oC$. The pulse, exposure, and purge times for the $MeCpPtMe_3$ were 0.5, 12, and 25 s, respectively, and for the $O_3$, 0.1, 12, and 25 s, respectively. $CoO_x$ nanoparticles were deposited at 250 $^oC$ with bis(cyclopentadienyl) cobalt ($Cp_2Co$) and $O_3$ as precursors. $Cp_2Co$ was kept at 70 $^oC$. The pulse, exposure, and purge times for the $Cp_2Co$ were 5.5, 16, and 25 s, respectively, and for the $O_3$, 0.1, 12, and 25 s, respectively. $FeO_x$ nanoparticles were deposited at 250 $^oC$ with ferrocene ($Fe(Cp)_2$) and $O_3$ as precursors. $Fe(Cp)_2$ was kept at 90 $^oC$. The pulse, exposure, and purge times for the $Fe(Cp)_2$ were 0.8, 8, and 20 s, respectively, and for the $O_3$, 0.1, 12, and 25 s, respectively. The $Al_2O_3$ film was deposited at 125 $^oC$ with trimethylaluminum (TMA) and deionized $H_2O$ as precursors. The pulse, exposure, and purge times for the TMA were 0.02, 8, and 25 s, respectively, and for the $H_2O$, 0.1, 8, and 25 s, respectively.

**Catalyst characterizations.** TEM and HRTEM images were acquired with a JEOL-2100F field-emission transmission electron microscope operated at 200 kV. HAADF-STEM images and EDS mapping profiles were collected on a JEOL ARM-200F field-emission transmission electron microscope operated at 200 kV. The contents of Pt and Co metal in the catalysts were determined by ICP-AES. XRD patterns were collected on a Bruker D8 Advance X-ray diffractometer using a Cu Kα source (λ = 1.540 Å) in the $2\theta$ range from 10 °to 90 °. XPS data were taken on an ES-300 photoelectron spectrometer (KRATOS Analytical) using an Al Kα source (1486.6 eV). $N_2$ physical adsorption was carried out on Micromeritics ASAP2020 volumetric adsorption analyzer. Before the sorption measurements, samples were degassed at 423 K for 2 h. $H_2$-TPR experiments were performed with an Auto-Chem 2920 instrument, using a 50 mg sample under 50 ml·min$^{-1}$ $H_2$ (10%)/Ar flow with a heating rate of 10 ℃·min$^{-1}$ from room temperature to 800 ℃.

A mixture containing 20 mg of $WO_3$ and various catalysts was placed in a quartz reaction tube. Then the tube was placed in an oven with the temperature controlled to be 25 ℃. Then the hydrogen was allowed to flow through the reaction tube at a rate of 50 mL min$^{-1}$. Any color changes of the powder samples with the time were observed in the process.

## Supplementary Figures

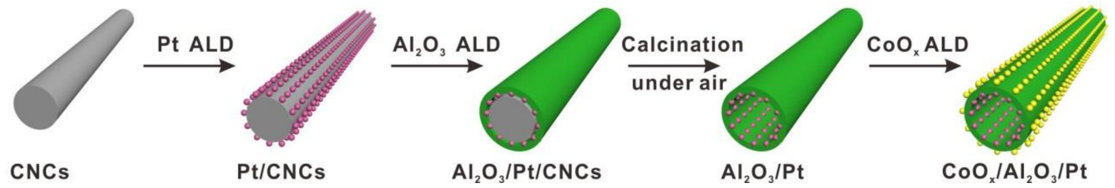


**Supplementary Figure 1. Schematic illustration of the preparation process of $CoO_x$/y$Al_2O_3$/Pt** (y is the cycle numbers of ALD $Al_2O_3$).

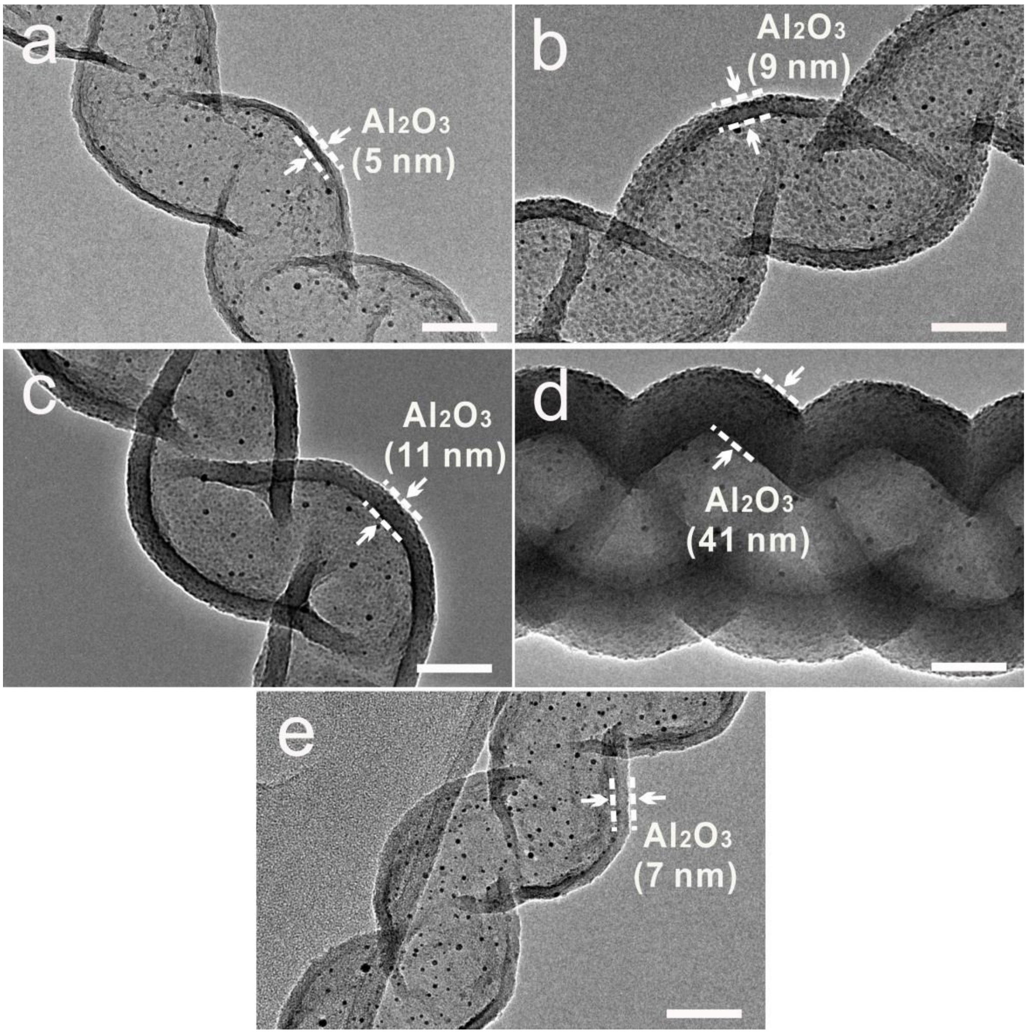


**Supplementary Figure 2. Structural characterization of the catalysts** (scale bar, 50 nm). TEM images of (**a**) $CoO_x/35Al_2O_3/Pt$, (**b**) $CoO_x/65Al_2O_3/Pt$, (**c**) $CoO_x/80Al_2O_3/Pt$, (**d**) $CoO_x/300Al_2O_3/Pt$, and (**e**) $50Al_2O_3/Pt$.

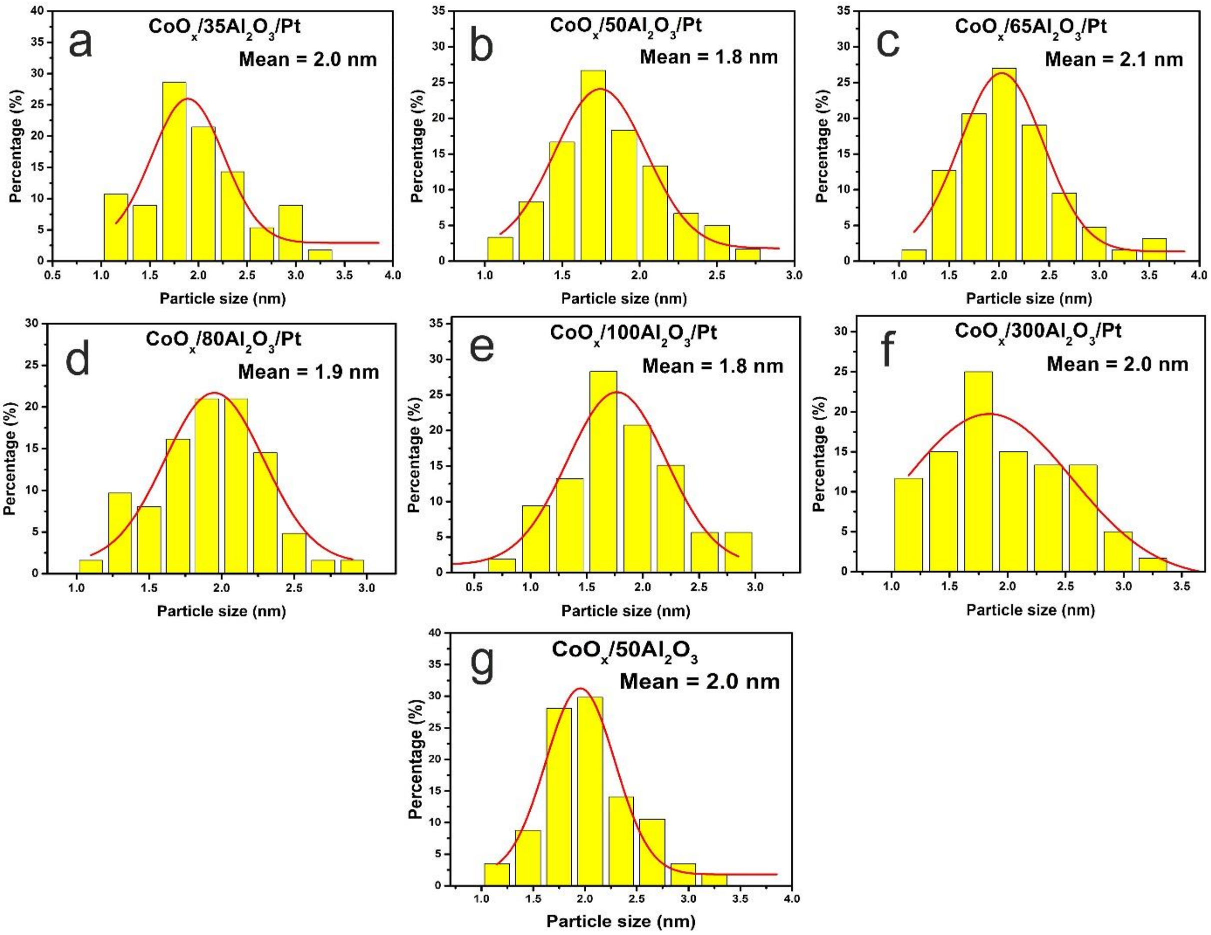


**Supplementary Figure 3. The size distributions of $CoO_x$ nanoparticles in the catalysts**. (**a**) $CoO_x/35Al_2O_3/Pt$, (**b**) $CoO_x/50Al_2O_3/Pt$, (**c**) $CoO_x/65Al_2O_3/Pt$, (**d**) $CoO_x/80Al_2O_3/Pt$, (**e**) $CoO_x/100Al_2O_3/Pt$, (**f**) $CoO_x/300Al_2O_3/Pt$, and (**g**) $CoO_x/50Al_2O_3$.

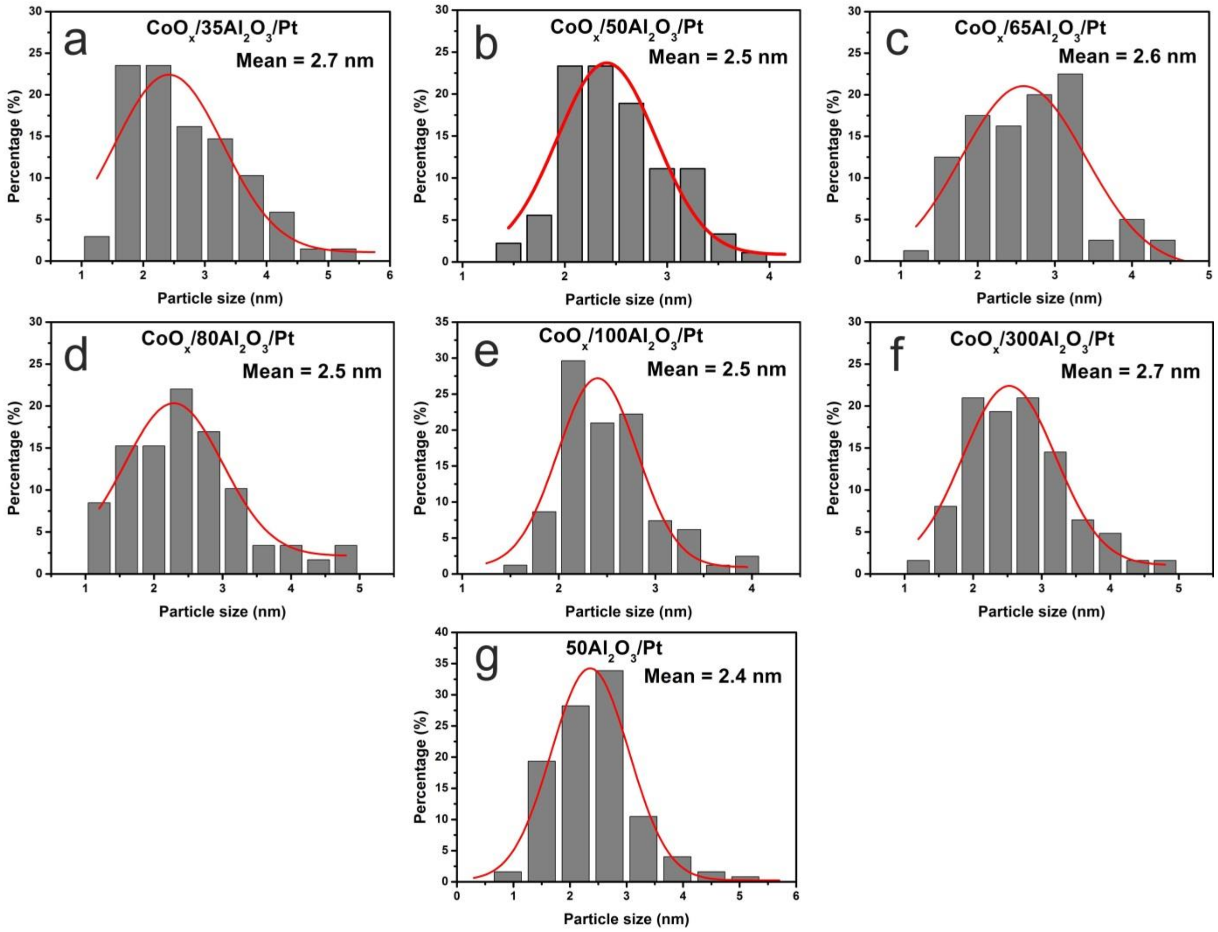


**Supplementary Figure 4. The size distributions of Pt nanoparticles in the catalysts**. (**a**) $CoO_x/35Al_2O_3/Pt$, (**b**) $CoO_x/50Al_2O_3/Pt$, (**c**) $CoO_x/65Al_2O_3/Pt$, (**d**) $CoO_x/80Al_2O_3/Pt$, (**e**) $CoO_x/100Al_2O_3/Pt$, (**f**) $CoO_x/300Al_2O_3/Pt$, and (**g**) $50Al_2O_3/Pt$.

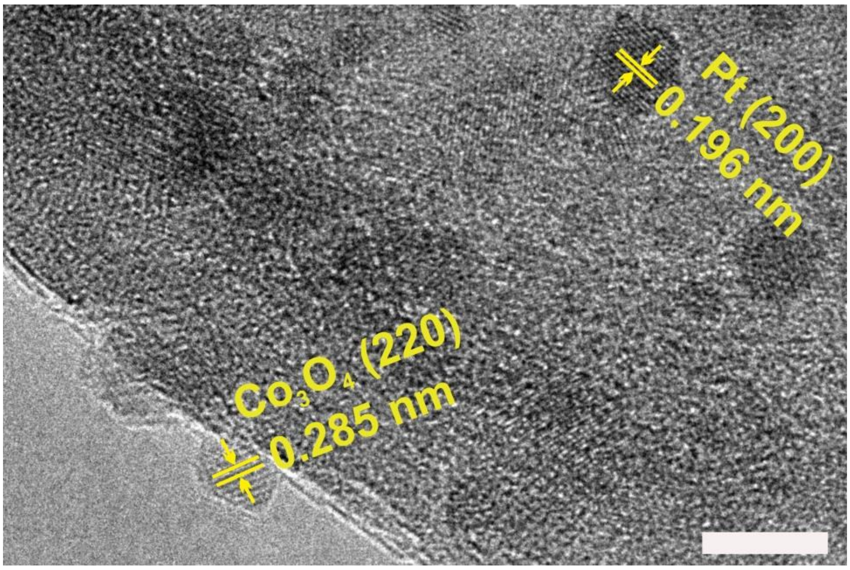


**Supplementary Figure 5. HRTEM image of $CoO_x$/50$Al_2O_3$/Pt** (scale bar, 5 nm). The measured distances between adjacent lattice fringes of the inner Pt nanoparticle is 0.196 nm and it is 0.285 nm for the outer $CoO_x$ nanoparticle, which correspond well with the lattice spacing of the Pt (200) planes and the $Co_3O_4$ (220) planes, respectively.

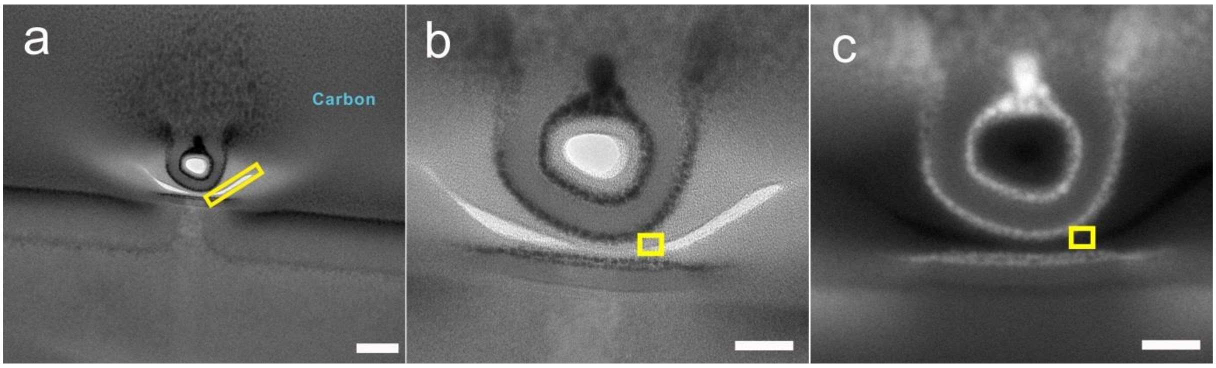


**Supplementary Figure 6. The cross-sectional TEM images of $CoO_x/50Al_2O_3/Pt$.** (**a-c**) TEM images of the cross-sectional specimen prepared by FIB (scale bar: a 50 nm; b-c 20 nm). Carbon was deposited on the sample as a protecting layer to enhance its stability. There are gaps in (**a**) (yellow rectangle), because part of the original CNC template is suspended when it is dispersed on the silicon substrate. The upper edge of the cross-sectional specimen is partially destroyed possibly during the ion milling process.

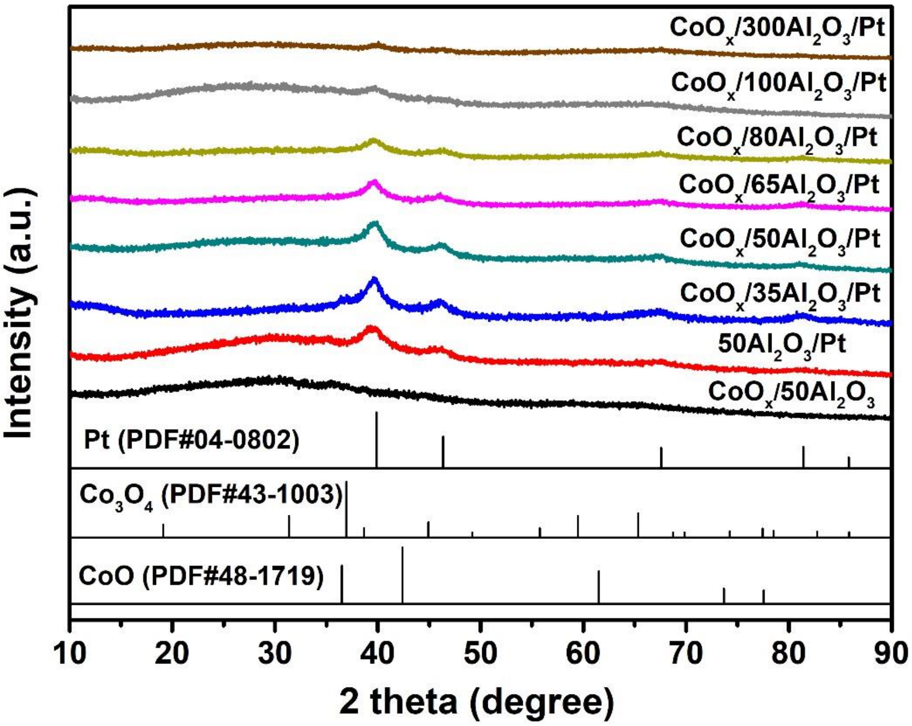


**Supplementary Figure 7. XRD patterns of different catalysts.** Four diffraction peaks positioned at 39.7°, 46.2°, 67.5° and 81.3° are observed, which can be ascribed to (111), (200), (220) and (311) crystal planes of face-centered cubic $Pt^0$ (JCPDS No.65-2868), respectively. The diffraction peaks of $Al_2O_3$ are not found, indicating that the $Al_2O_3$ layer is amorphous.

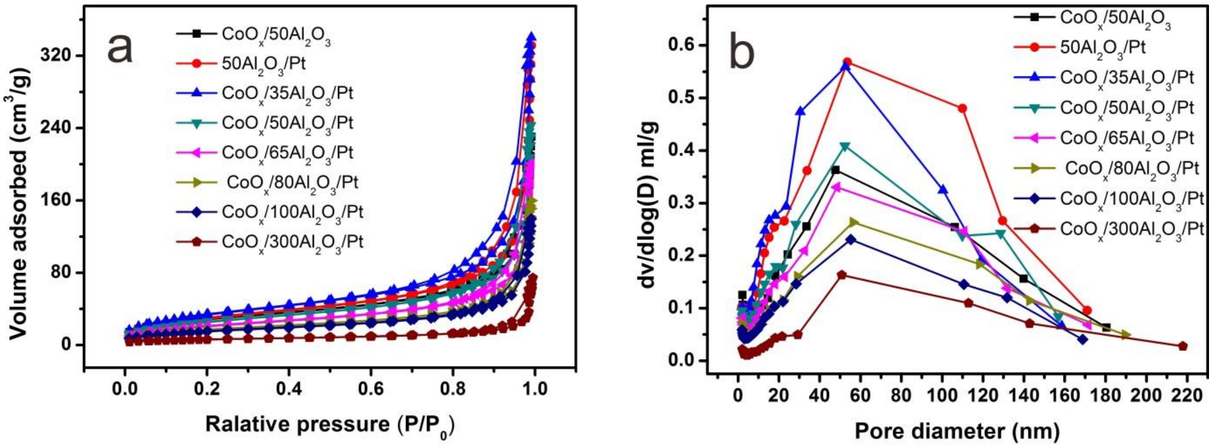


**Supplementary Figure 8. The pore structure characterization of the catalysts.** (**a**) The $N_2$ adsorption-desorption isotherms and (**b**) the corresponding pore size distributions of the catalysts. Brunauer-Emmett-Teller (BET) surface areas, pore volumes and average pore diameters of all the catalysts are listed in Supplementary Table 3.

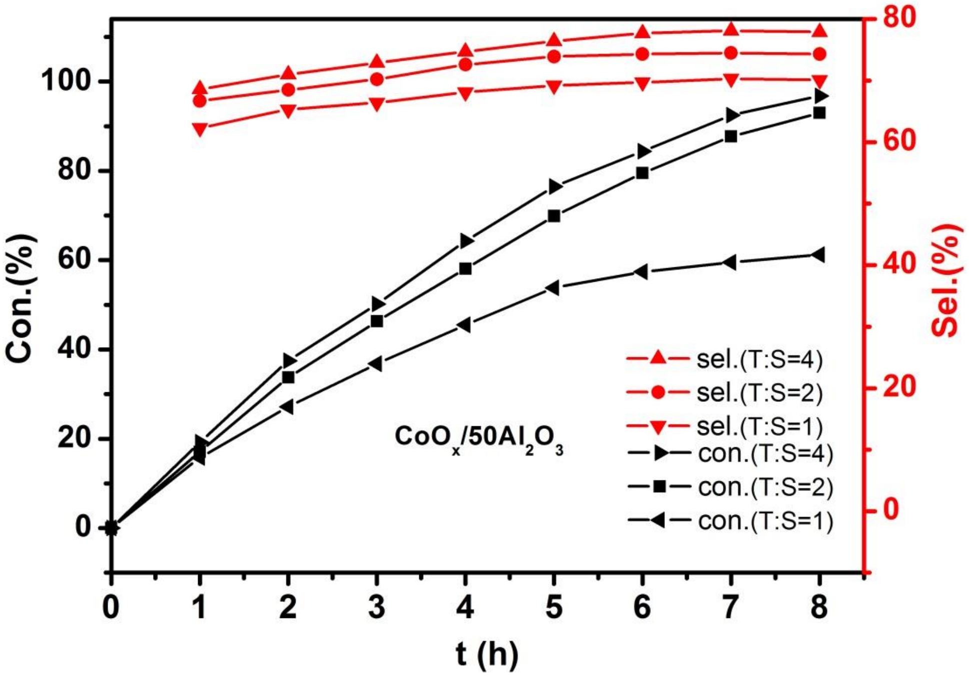


**Supplementary Figure 9. Catalytic performances of $CoO_x/50Al_2O_3$ for different mole ratio of TBHP to styrene.** T represents TBHP and S represents styrene. Reaction condition: styrene (3.5 mmol), acetonitrile (20 ml), catalyst (15 mg) and temperature (80 ℃). For $CoO_x/50Al_2O_3$, when the mole ratio of TBHP to styrene was decreased from 4 to 1, both the activity and SO selectivity were reduced. This implys that a decrease in activity does not necessarily lead to an increase in selectivity, which may also be decreased. Thus, it can be concluded that the enhanced epoxidation selectivity by introducing controllable hydrogen spillover into the reaction was not due to the decrease of activity.

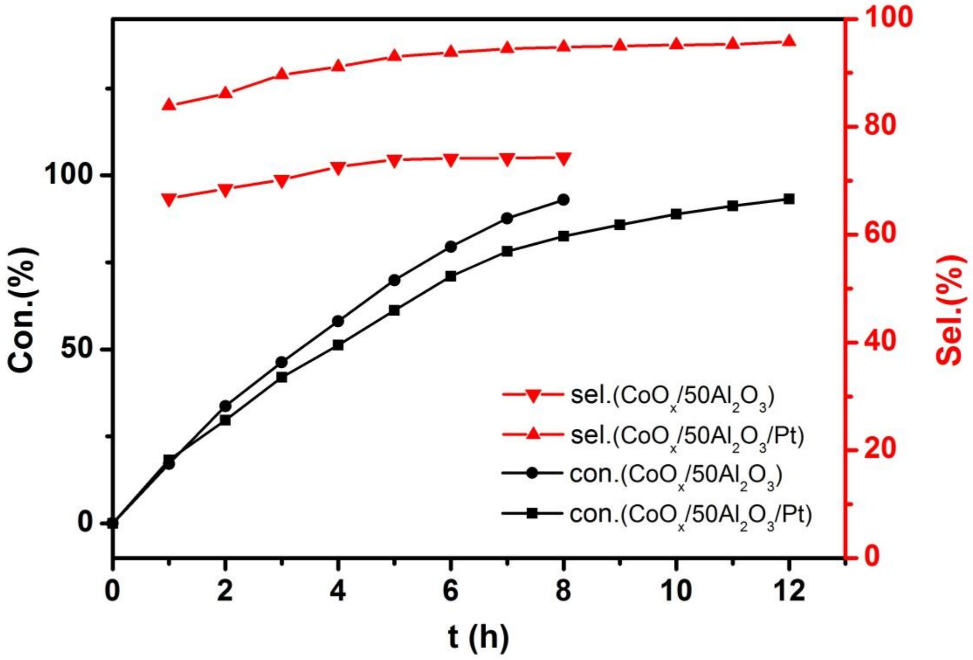


**Supplementary Figure 10. Catalytic performance of $CoO_x/50Al_2O_3$ and $CoO_x/50Al_2O_3/Pt$.** The evolution of styrene conversion and SO selectivity with reaction time over $CoO_x/50Al_2O_3$ (in the TBHP condition) and $CoO_x/50Al_2O_3/Pt$ (in the $H_2$-TBHP condition).

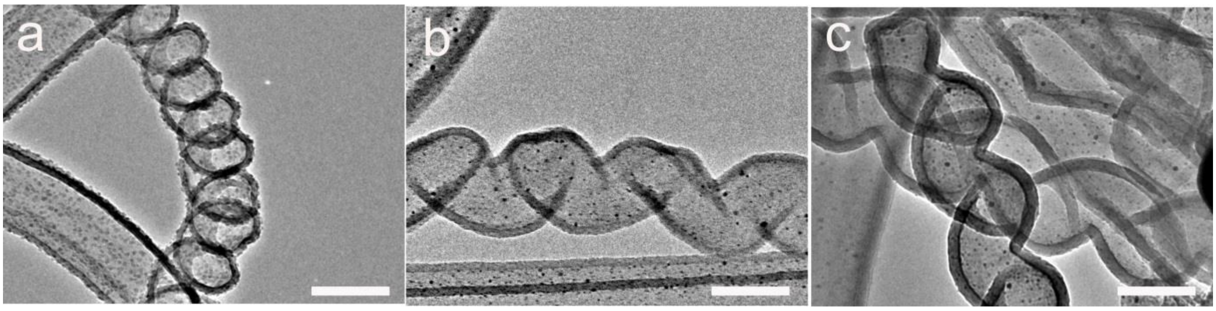


**Supplementary Figure 11. Structural characterization of the used catalysts.** TEM images (scale bar, 100 nm) of (**a**) $CoO_x/50Al_2O_3$, (**b**) $CoO_x/50Al_2O_3/Pt$, and (**c**) $CoO_x/100Al_2O_3/Pt$ after reaction.

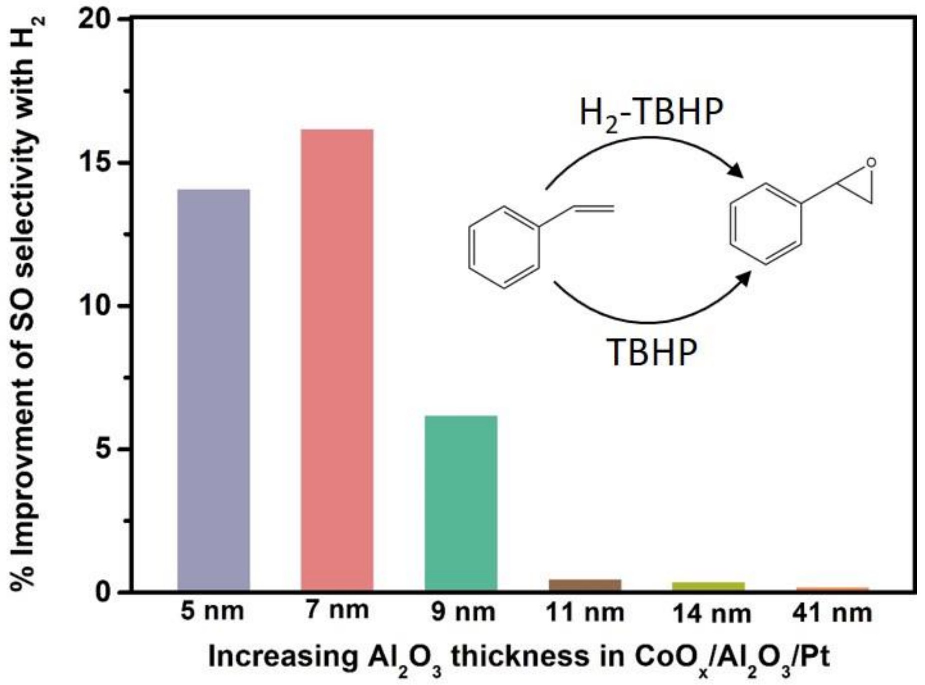


**Supplementary Figure 12. Percent improvement of SO selectivity for $CoO_x$/$yAl_2O_3$/Pt with different $Al_2O_3$ thicknesses after 1 h reaction.**

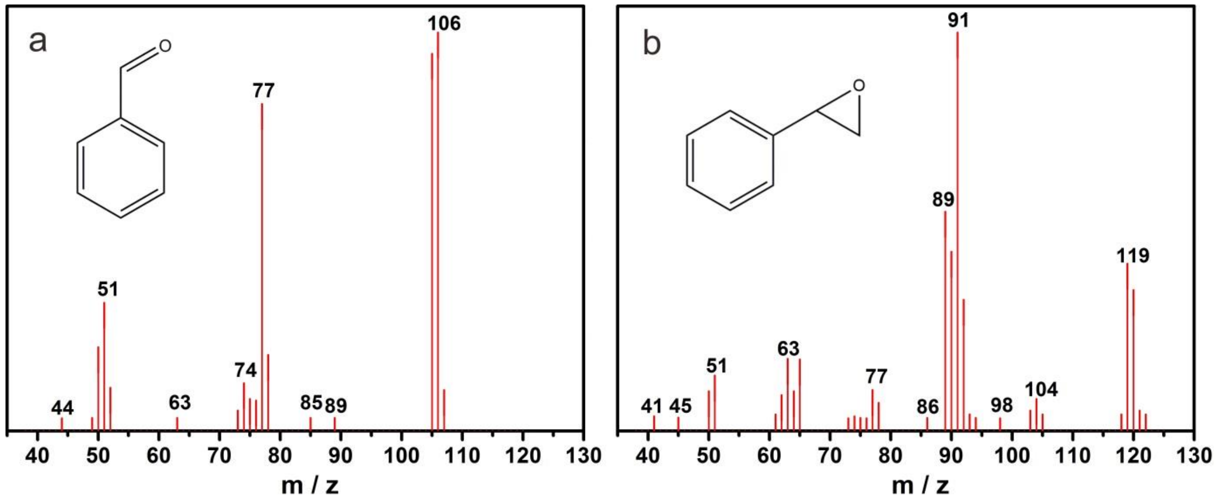


**Supplementary Figure 13. Mass spectrometry of products.** Mass spectrometry of (a) benzaldehyde and (b) styrene oxide over $CoO_x/50Al_2O_3/Pt$ catalyst (in the $D_2$-TBHP condition).

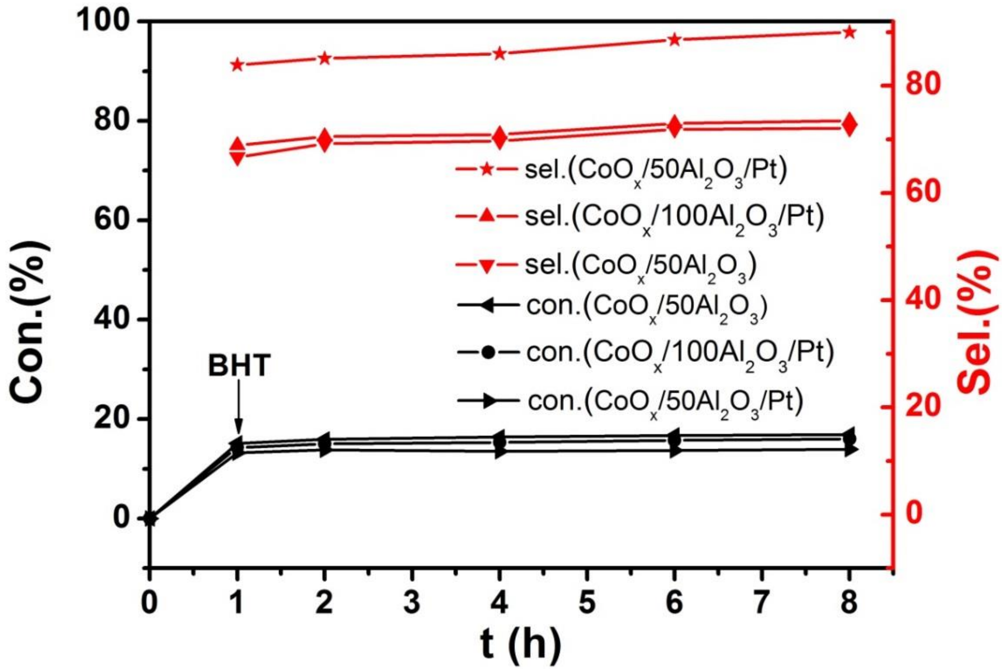


**Supplementary Figure 14. Radical quenching experiments.** The evolution of styrene conversion and SO selectivity for $CoO_x/50Al_2O_3$, $CoO_x/50Al_2O_3/Pt$, and $CoO_x/100Al_2O_3/Pt$ with reaction time after the BHT addition.

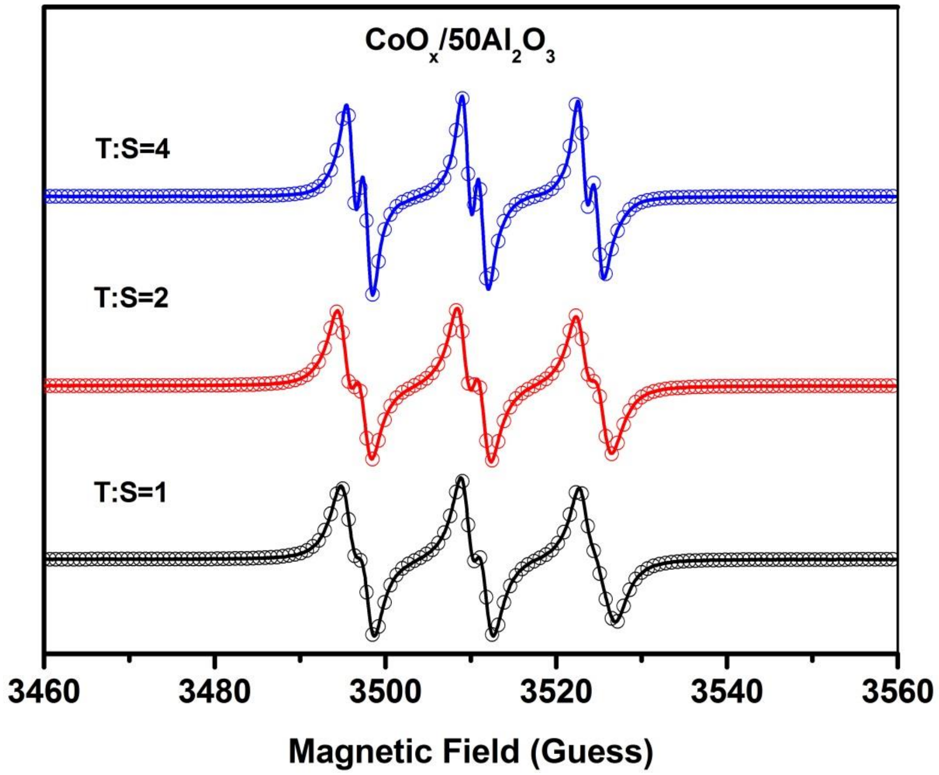


**Supplementary Figure 15. EPR characterization.** Experimental EPR spectra (lines) for $CoO_x/50Al_2O_3$ in the TBHP condition with different molar ratio of TBHP to styrene (T represents TBHP; S represents styrene) and simulated spectra (open circles) for a mixture of PBN-OOC$(CH_3)_3$ and PBN-OC$(CH_3)_3$. Hyperfine coupling constants of PBN-OOC$(CH_3)_3$ ($AG_N$ = 14.42 and $AG_H$ = 2.18) and PBN-OC$(CH_3)_3$ ($AG_N$ = 13.52 and $AG_H$ = 1.72).

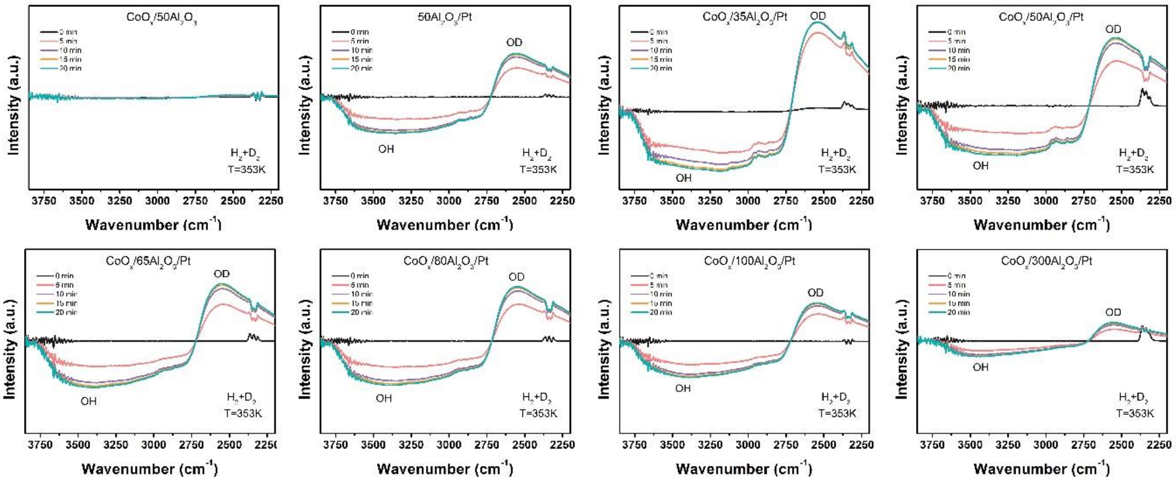


**Supplementary Figure 16. H-D exchange experiments.** IR spectra of the catalysts during H-D exchange in a stream of $H_2$+$D_2$ at 353K and 1 atm (flow rates of $H_2$ and of $D_2$, each 15ml (NTP) $min^{-1}$). For $CoO_x/50Al_2O_3$, no υ(OD) band appeared, indicating no H-D transformation occurred. For all the Pt-containing catalysts, during this exchange process, the υ(OH) band at 3000-3700 $cm^{-1}$ was diminishing, while the intensity of the υ(OD) band at 2378-2724 $cm^{-1}$ was increasing with time. When the thicknesses of $Al_2O_3$ layer are less than 11 nm ($80Al_2O_3$), the addtion of $CoO_x$ may promote H-D exchange compared to $50Al_2O_3$/Pt. With a further increase of thicknesses (over 11 nm), H-D exchange rates of $CoO_x/100Al_2O_3$/Pt and $CoO_x/300Al_2O_3$/Pt are lower than that of $50Al_2O_3$/Pt, demonstrating that remote $CoO_x$ has negligible contribution on H-D exchange rates.

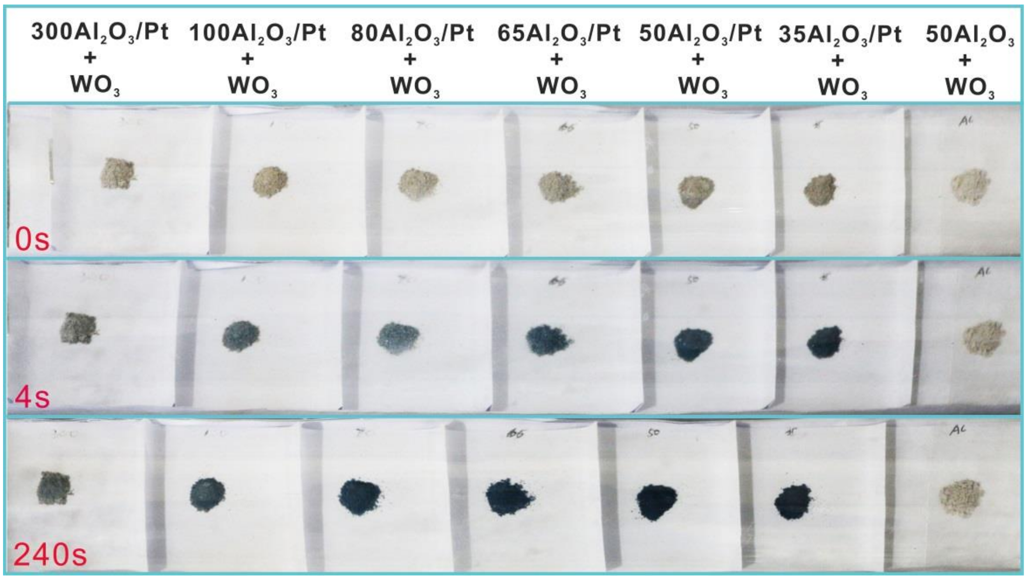


**Supplementary Figure 17. Color change photographs of the mixtures.** Photographs of samples made with $WO_3$ mixed with the catalysts before treatment (0s) and after treatment with 5% $H_2$/Ar at 25 ℃ for 4s and 240 s. The mixture of 50$Al_2O_3$ and $WO_3$ exhibited an unchanged color. The mixture 35$Al_2O_3$/Pt and $WO_3$ gave a dark blue color, which was because that the spilled-over hydrogen migrates and readily reacts with $WO_3$ to form dark blue $H_xWO_3$[1]. With incerasing the thicknesses of $Al_2O_3$ layer from 5 nm to 41 nm, the color became more lighter.

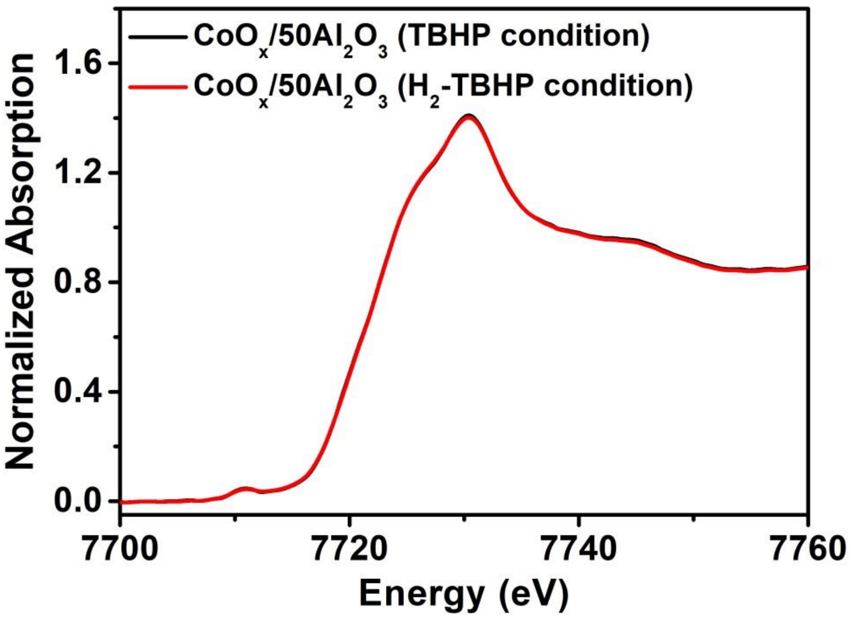


**Supplementary Figure 18. In situ XAFS characterization.** In situ Co K-edge XANES spectra of $CoO_x/50Al_2O_3$ in the TBHP and $H_2$-TBHP condition.

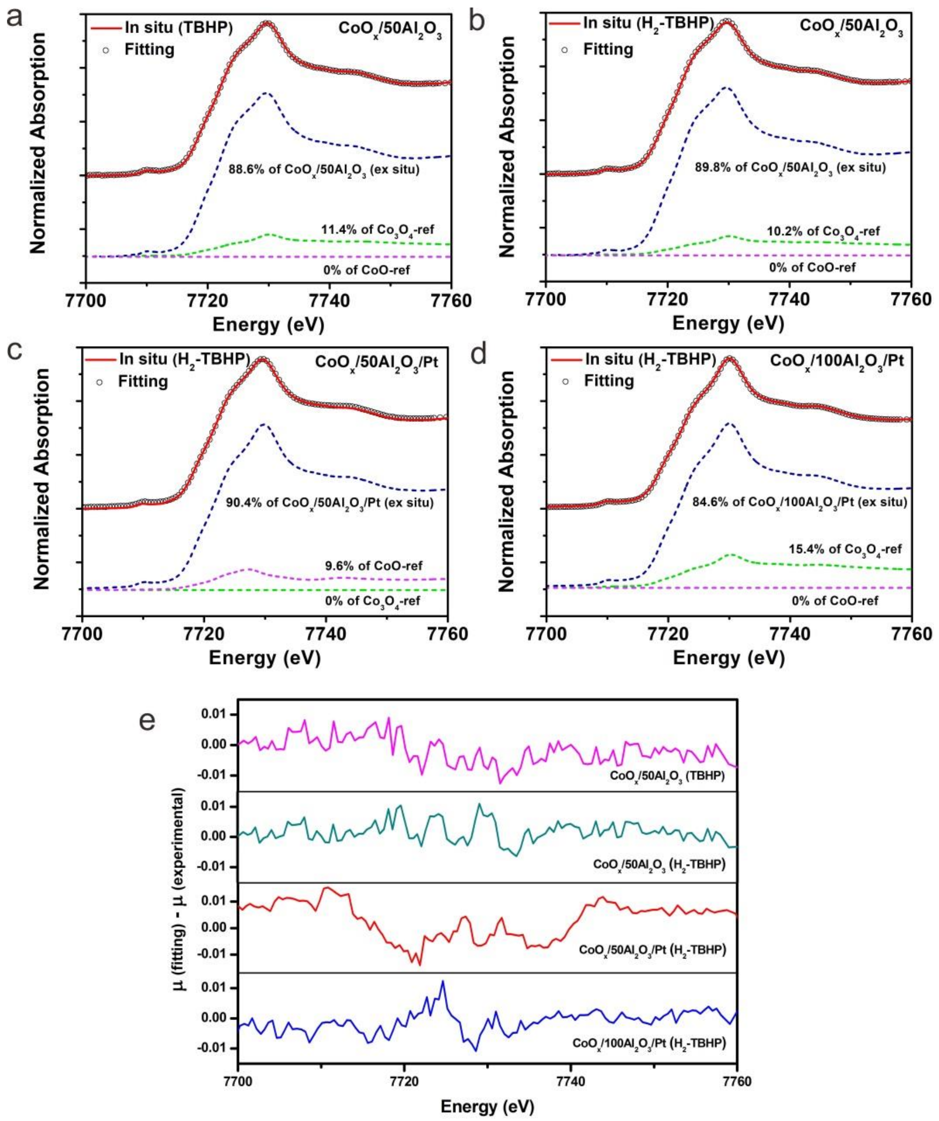


**Supplementary Figure 19. Linear combination fitting**. (**a-d**) In situ XANES spectra of (**a, b**) $CoO_x/50Al_2O_3$, (**c**) $CoO_x/50Al_2O_3/Pt$, (**d**) $CoO_x/100Al_2O_3/Pt$ with their linear combination fitting results. For each catalyst, the in-situ XANES spectrum was simulated by a linear combination of the ex-situ spectrum of the as-prepared catalyst and the spectra of reference samples ($Co_3O_4$ and CoO). (**e**) The difference plots between the fits and data for $CoO_x/50Al_2O_3$, $CoO_x/50Al_2O_3/Pt$ and $CoO_x/100Al_2O_3/Pt$ (subtracting the experimental spectrum from the fitting spectrum).

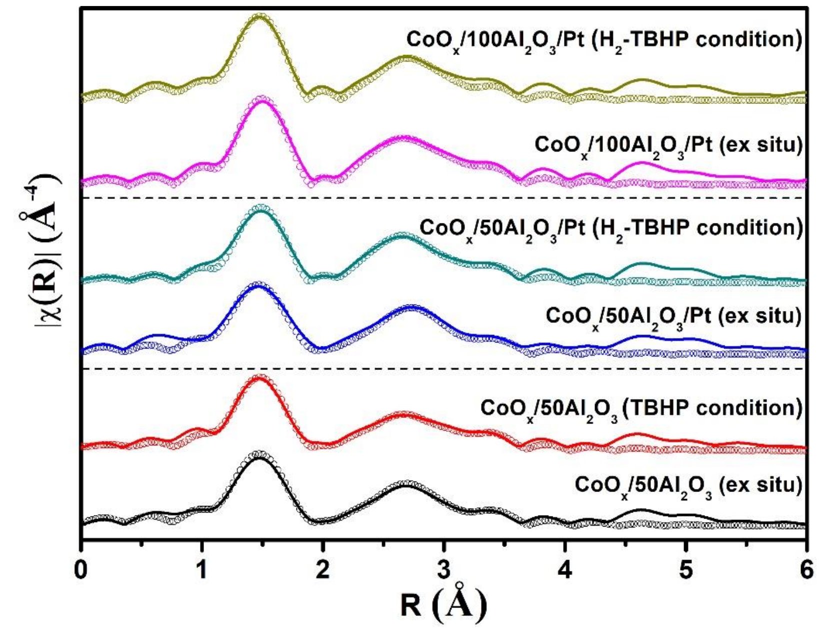


**Supplementary Figure 20. EXAFS spectra of the catalysts.** Fourier transforms (FTs) of EXAFS spectra collected at the cobalt K edge of $CoO_x/50Al_2O_3$, $CoO_x/50Al_2O_3/Pt$ and $CoO_x/100Al_2O_3/Pt$, respectively. Lines indicate experimental data and open circles indicate fitted results.

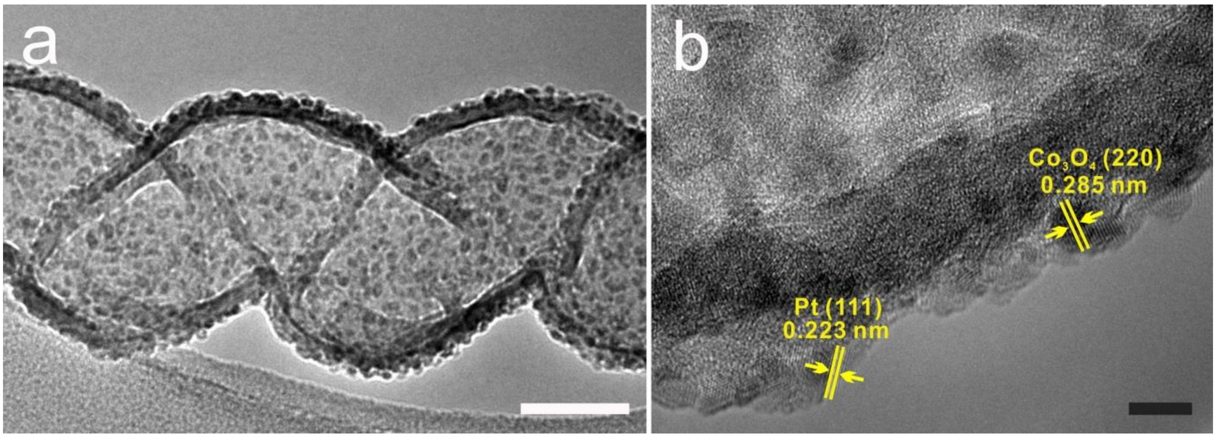


**Supplementary Figure 21. TEM characterization of $CoO_xPt/50Al_2O_3$.** (**a**) TEM image (scale bar, 50 nm). (**b**) HRTEM image (scale bar, 5 nm)

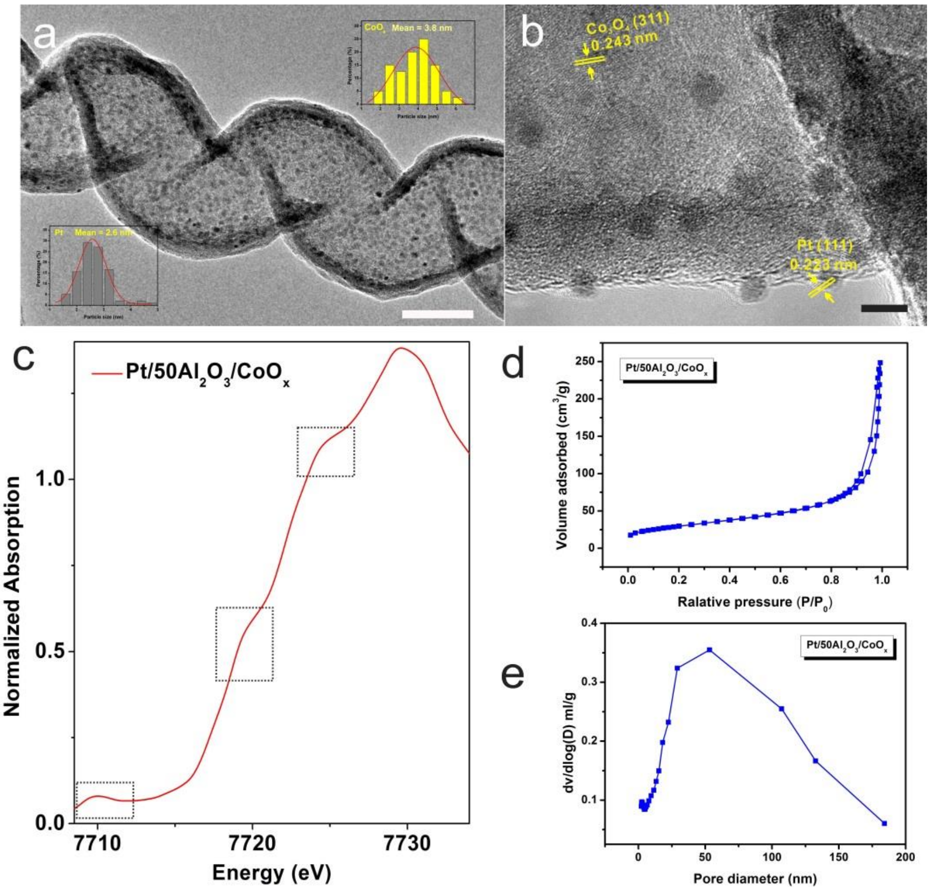


**Supplementary Figure 22. Structure characterization of $Pt/50Al_2O_3/CoO_x$.** (**a**) TEM image (scale bar, 50 nm) and the size distributions of Pt and $CoO_x$ nanoparticles (insets); (**b**) HRTEM image (scale bar, 5 nm); (**c**) XAFS spectrum; The characteristic line shapes of these positions (7709 eV, 7719 eV and 7724 eV) indicate the formation of $CoAlO_x$.[2] (**d**) The $N_2$ adsorption-desorption isotherms and (**e**) the corresponding pore size distribution.

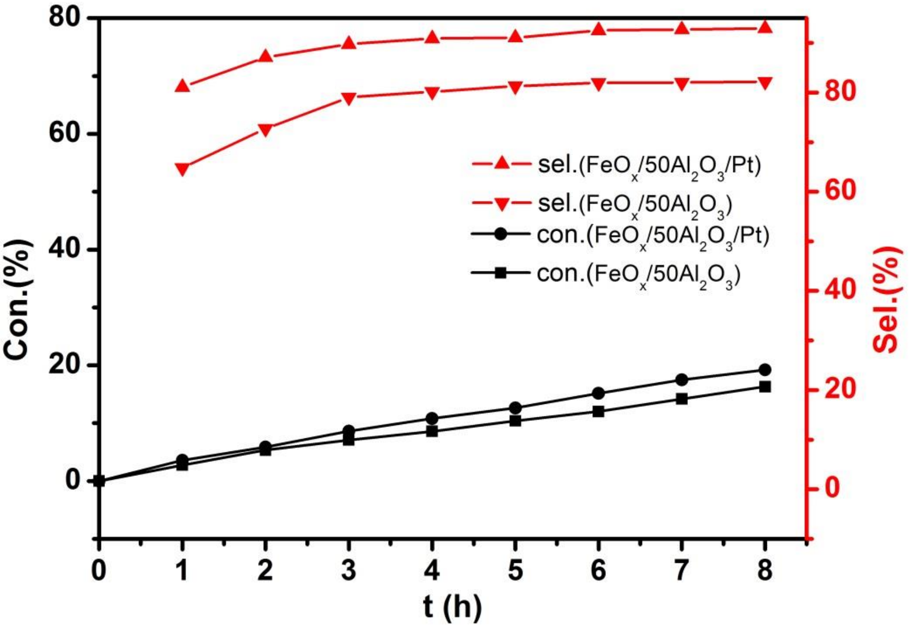


**Supplementary Figure 23. Catalytic performance of $FeO_x/50Al_2O_3$ and $FeO_x/50Al_2O_3/Pt$.** The evolution of styrene conversion and SO selectivity with reaction time over $FeO_x/50Al_2O_3$ (in the TBHP condition) and $FeO_x/50Al_2O_3/Pt$ (in the $H_2$-TBHP condition).

## Supplementary Tables

**Supplementary Table 1. The sizes of Pt nanoparticles in $CoO_x/yAl_2O_3/Pt$ resulted from TEM and XRD.**

| Catalysts | Distance (nm) | Pt particle size (nm) | |
|---|---|---|---|
| | | TEM[a] | XRD[b] |
| $50Al_2O_3/Pt$ | - | 2.4 | 3.9 |
| $CoO_x/35Al_2O_3/Pt$ | 5 | 2.7 | 3.8 |
| $CoO_x/50Al_2O_3/Pt$ | 7 | 2.5 | 4.2 |
| $CoO_x/65Al_2O_3/Pt$ | 9 | 2.6 | 4.3 |
| $CoO_x/80Al_2O_3/Pt$ | 11 | 2.5 | 4.4 |
| $CoO_x/100Al_2O_3/Pt$ | 14 | 2.5 | - |
| $CoO_x/300Al_2O_3/Pt$ | 41 | 2.7 | - |

[a] Pt particle size determined by TEM.

[b] Pt particle size calculated from XRD using the most intense peak located at $2\theta = 39.7°$.

**Supplementary Table 2. Loading amounts of Pt and Co for the catalysts.**

| Sample[a] | Pt content (wt.%) | Co content (wt.%) |
|---|---|---|
| $CoO_x/50Al_2O_3$ | - | 4.39 |
| $50Al_2O_3/Pt$ | 4.23 | - |
| $CoO_x/35Al_2O_3/Pt$ | 4.05 | 3.89 |
| $CoO_x/50Al_2O_3/Pt$ | 3.94 | 3.70 |
| $CoO_x/65Al_2O_3/Pt$ | 3.05 | 2.98 |
| $CoO_x/80Al_2O_3/Pt$ | 2.85 | 2.41 |
| $CoO_x/100Al_2O_3/Pt$ | 1.92 | 1.56 |
| $CoO_x/300Al_2O_3/Pt$ | 0.68 | 0.52 |
| $Pt/50Al_2O_3/\ CoO_x$ | 1.63 | 4.77 |

[a] The ALD cycles of Pt and $CoO_x$ are 20 and 35, respectively.

**Supplementary Table 3. The specific surface area and pore structure parameters of the catalysts**.

| Catalysts | BET surface area ($m^2$ $g^{-1}$) | Pore volume ($cm^3$ $g^{-1}$) | Average pore diameter (nm) |
|---|---|---|---|
| $CoO_x/50Al_2O_3$ | 103.8 | 0.36 | 47.8 |
| $50Al_2O_3/Pt$ | 110.0 | 0.51 | 53.3 |
| $CoO_x/35Al_2O_3/Pt$ | 124.7 | 0.53 | 52.1 |
| $CoO_x/50Al_2O_3/Pt$ | 94.5 | 0.38 | 52.5 |
| $CoO_x/65Al_2O_3/Pt$ | 77.0 | 0.31 | 48.2 |
| $CoO_x/80Al_2O_3/Pt$ | 62.5 | 0.25 | 56.1 |
| $CoO_x/100Al_2O_3/Pt$ | 55.5 | 0.22 | 54.8 |
| $CoO_x/300Al_2O_3/Pt$ | 22.2 | 0.12 | 50.9 |
| $Pt/50Al_2O_3/$ $CoO_x$ | 105.3 | 0.38 | 53.2 |

**Supplementary Table 4. The reduction peak area and the degree of reduction of Co and Pt for the catalysts from TPR**.

| Catalysts | Reduction peak temperature ($^{o}C$) | Peak area | Total area | Degree of reduction (%) | |
|---|---|---|---|---|---|
| | | | | Co | Pt |
| $50Al_2O_3/Pt$ | 116 | 163.5 | 3600.6 | - | 100 |
| | 386 | 3437.1 | | | |
| $CoO_x/50Al_2O_3$ | 320 | 467.3 | 1612.3 | 6.1 | - |
| | 442 | 320.9 | | | |
| | 627 | 824.1 | | | |
| $CoO_x/50Al_2O_3/Pt$ | 112 | 251.8 | 5907.7 | 8.6 | 100 |
| | 216 | 377.7 | | | |
| | 365 | 4848.2 | | | |
| | 622 | 430.0 | | | |
| $CoO_x/100Al_2O_3/Pt$ | 361 | 4498.5 | 5281.0 | 6.9 | 100 |
| | 626 | 782.5 | | | |

**Supplementary Table 5. The carbon balance results**.

| Catalysts | Carbon balance of styrene (%) | |
|---|---|---|
| | TBHP condition | $H_2$-TBHP condition |
| $CoO_x/35Al_2O_3/Pt$ | 99.0 | 99.2 |
| $CoO_x/50Al_2O_3/Pt$ | 98.2 | 99.4 |
| $CoO_x/65Al_2O_3/Pt$ | 98.5 | 100.3 |
| $CoO_x/80Al_2O_3/Pt$ | 97.8 | 97.8 |
| $CoO_x/100Al_2O_3/Pt$ | 99.0 | 98.5 |
| $CoO_x/300Al_2O_3/Pt$ | 100.2 | 98.2 |

**Supplementary Table 6. The EPR fitting results.**

| Atmosphere | Catalysts | T:S | PBN-tBuO | PBN-tBuOO | tBuOO·/ tBuO· |
|---|---|---|---|---|---|
| - | $CoO_x/50Al_2O_3$ | 1 | $1.697\times10^{15}$ | $2.602\times10^{15}$ | 1.53 |
| - | $CoO_x/50Al_2O_3$ | 2 | $2.523\times10^{15}$ | $2.129\times10^{15}$ | 0.84 |
| - | $CoO_x/50Al_2O_3$ | 4 | $2.244\times10^{15}$ | $9.555\times10^{14}$ | 0.43 |
| $H_2$ | $CoO_x/50Al_2O_3/Pt$ | 2 | $3.013\times10^{14}$ | $1.031\times10^{15}$ | 3.42 |
| $H_2$ | $CoO_x/100Al_2O_3/Pt$ | 2 | $3.188\times10^{14}$ | $6.044\times10^{14}$ | 1.89 |

**Supplementary Table 7. Linear combination fitting results of in situ XANES spectra**.

| Catalysts | Condition | $f_1$ | $f_2$ | $f_3$ |
|---|---|---|---|---|
| $CoO_x/50Al_2O_3$ | TBHP | 0.886±0.005 | 0.114±0.005 | 0.000±0.008 |
| $CoO_x/50Al_2O_3$ | $H_2$-TBHP | 0.898±0.004 | 0.102±0.006 | 0.000±0.007 |
| $CoO_x/50Al_2O_3/Pt$ | $H_2$-TBHP | 0.904±0.007 | 0.000±0.005 | 0.096±0.006 |
| $CoO_x/100Al_2O_3/Pt$ | $H_2$-TBHP | 0.846±0.009 | 0.154±0.009 | 0.000±0.013 |

**Supplementary Table 8. FT-EXAFS parameters of $Co_3O_4$, $CoO_x/50Al_2O_3$, $CoO_x/50Al_2O_3/Pt$ and $CoO_x/100Al_2O_3/Pt$.**

| Sample | shell | R (Å) | CN | $\Delta\sigma^2 \times 10^3$ (Å$^2$) | $\Delta E_0$ (eV) |
|---|---|---|---|---|---|
| $Co_3O_4$ | Co-O | 1.92 | 5.33 | - | - |
| | Co-$Co_1$ | 2.82 | 4 | - | - |
| | Co-$Co_2$ | 3.33 | 8 | - | - |
| $CoO_x/50Al_2O_3$ | Co-O | 1.92 | 2.9 | 6.2 | 8.5 |
| | Co-$Co_1$ | 2.85 | 1.7 | 3.7 | 12.3 |
| | Co-$Co_2$ | 3.37 | 5.6 | 8.5 | 12.3 |
| $CoO_x/50Al_2O_3$-in situ | Co-O | 1.92 | 3.0 | 6.4 | 7.6 |
| | Co-$Co_1$ | 2.86 | 1.6 | 4.8 | 14.0 |
| | Co-$Co_2$ | 3.39 | 3.8 | 5.2 | 14.0 |
| $CoO_x/50Al_2O_3/Pt$ | Co-O | 1.92 | 2.6 | 3.9 | 8.3 |
| | Co-$Co_1$ | 2.89 | 1.9 | 4.3 | 13.0 |
| | Co-$Co_2$ | 3.40 | 4.9 | 5.7 | 13.0 |
| $CoO_x/50Al_2O_3/Pt$-in situ | Co-O | 1.92 | 2.6 | 7.3 | 8.5 |
| | Co-$Co_1$ | 2.89 | 1.9 | 6.4 | 14.5 |
| | Co-$Co_2$ | 3.40 | 5.0 | 6.8 | 14.5 |
| $CoO_x/100Al_2O_3/Pt$ | Co-O | 1.91 | 3.5 | 3.4 | 9.2 |
| | Co-$Co_1$ | 2.82 | 1.9 | 1.9 | 8.9 |
| | Co-$Co_2$ | 3.33 | 6.4 | 7.8 | 8.9 |
| $CoO_x/100Al_2O_3/Pt$-in situ | Co-O | 1.90 | 3.5 | 4.1 | 8.6 |
| | Co-$Co_1$ | 2.84 | 1.7 | 3.7 | 10.4 |
| | Co-$Co_2$ | 3.35 | 4.8 | 7.1 | 10.4 |

CN, coordination number; R, bonding distance; $\Delta\sigma^2$, Debye-Waller factor; $\Delta E_0$, inner potential shift.

**Supplementary Table 9. Catalytic performance of $CoO_xPt/50Al_2O_3$ for styrene epoxidation reaction for 8 h**.

| Catalysts | TBHP condition | | $H_2$-TBHP condition | |
|---|---|---|---|---|
| | Conversion (%) | Styrene oxide Sel. (%) | Conversion (%) | Styrene oxide Sel. (%) |
| $CoO_xPt/50Al_2O_3$ | 92.8 | 76.1 | 84.2 | 92.1 |

**Supplementary Table 10. Catalytic performance of Pt/50$Al_2O_3$/$CoO_x$ for styrene epoxidation reaction.**

| Atmosphere | Conversion (%) | Styrene oxide Sel.(%) | Yield (%) |
|---|---|---|---|
| - | 72.4 | 77.5 | 56.1 |
| $H_2$ | 60.3 | 84.5 | 51.0 |

## Supplementary Notes

**Note to Supplementary Table 1.** From XRD results, the average diameters of Pt nanoparticles for $CoO_x/yAl_2O_3/Pt$ with different $Al_2O_3$ thicknesses (5 nm, 7nm, 9 nm, 11 nm) are similar. For $CoO_x/100Al_2O_3/Pt$ and $CoO_x/300Al_2O_3/Pt$, the average diameters of Pt nanoparticles failed to be calucated by Scherrer formula, because the weak diffraction peaks of Pt nanoparticles in XRD patterns can result in great calculated error.

**Note to Supplementary Table 4.** The $Co_3O_4$ exhibits two peaks centred at 342 ℃ and 402 ℃, corresponding to the reductions of $Co_3O_4$ and CoO, respectively. The $CoO_x/50Al_2O_3$ exhibits two weak peaks centred at 320 ℃ and 442 ℃, and a strong peak centred at 627 ℃, corresponding to the reductions of $Co_3O_4$, CoO, and the $CoO_x$ interacting with $Al_2O_3$, respectively[3]. For $50Al_2O_3/Pt$, a weak peak appears at 116 ℃, and a principal peak is located at 386 ℃, which are attributed to reduction of the oxidized Pt species and Pt interacting with $Al_2O_3$, respectively[4-5]. For $CoO_x/50Al_2O_3/Pt$, one principal peak centred at 365 ℃ is due to the overlapping reduction peaks of CoO and Pt interacting with $Al_2O_3$. Three other peaks located at 112 ℃, 216 ℃ and 622 ℃ are associated with the reduction of the oxidized Pt species, $Co_3O_4$ and $CoO_x$ interacting with $Al_2O_3$, respectively. The $CoO_x/100Al_2O_3/Pt$ have a primary peak at 361 ℃, which originated from the overlapping of the three peaks (the reduction of $Co_3O_4$, CoO and Pt interacting with $Al_2O_3$), and a weak peak at approximately 626 ℃.

**Note to Supplementary Table 6.** Reaction condition: 0.36 mmol styrene, 0.72 mmol TBHP, 1:1 molar ratio between the spin trap (PBN) and TBHP, 5 ml acetonitrile and 2 mg catalyst at 80 ℃ (T represents TBHP; S represents styrene). When the mole ratio of TBHP to styrene was 2, among the three catalysts ($CoO_x/50Al_2O_3$ in the TBHP condition, $CoO_x/50Al_2O_3/Pt$ in the $H_2$-TBHP condition and $CoO_x/100Al_2O_3/Pt$ in the $H_2$-TBHP condition), the $CoO_x/50Al_2O_3/Pt$ in the $H_2$-TBHP condition exhibited the highest SO selectivity, and its ratio of tBuOO·/ tBuO· is also the highest. When the mole ratio of TBHP to styrene was increased from 1 to 4 , for $CoO_x/50Al_2O_3$ in the TBHP condition, SO selectivity showed an increase, while the ratio of tBuOO·/ tBuO· showed an decrease. The above results show that the enhanced SO selectivity cannot be simply ascribed to the increase of the tBuOO·/ tBuO· ratio.

**Note to Supplementary Table 10.** In the TBHP condition, the catalytic activity (72.4%) of $Pt/50Al_2O_3/CoO_x$ was obviously lower than that of $CoO_x/50Al_2O_3/Pt$ (93.4%), but the selectivities (77.5% and 76.2%) of the two catalysts were similar. This may due to the formation of composite oxides ($CoAlO_x$) during the calcination (Figure S21c), resulting in lower catalytic activity. Considering the different structures between $Pt/50Al_2O_3/CoO_x$ and $CoO_x/50Al_2O_3/Pt$, mass transport may also play a role for the lower catalytic activity of $Pt/50Al_2O_3/CoO_x$. When $H_2$ was introduced into the reaction, SO selectivity (84.5%) of $Pt/50Al_2O_3/CoO_x$ was increased by 7% compared to that in the TBHP condition, although its conversion (60.3%) was slightly reduced. However, in addition to styrene oxide and benzaldehyde, the additional hydrogenation by-product (ethylbenzene) was detected. This may be because that the weaker catalytic activity of composite oxides made styrene easier to be hydrogenated. Moreover, the composite oxides ($CoAlO_x$) are more difficult to be reduced than cobalt oxide[6], leading to non-efficient utilization of active hydrogen species. Therefore, the active hydrogen species may tend to undergo hydrogenation reaction.

## Supplementary References


1. Khoobiar, S. Particle to particle migration of hydrogen atoms on platinum-alumina catalysts from particle to neighboring particles. *J. Phys. Chem.* **68,** 411-412 (1964).
2. Moen, A., Nicholson, D., Clausen, B. S., Hansen, P. L., Molenbroek, A. M., & Steffensen, G. X-ray absorption spectroscopic studies at the cobalt K-edge on a reduced $Al_2O_3$-supported rhenium-promoted cobalt Fischer-Tropsch catalyst. *Chem. Mater.* **9,** 1241-1247 (1997).
3. Ji, Y., Zhao, Z., Duan, A., Jiang G. & Liu, J. Comparative study on the formation and reduction of bulk and $Al_2O_3$-supported cobalt oxides by $H_2$-TPR technique. *J. Phys. Chem. C,* **113,** 7186-7199 (2009).
4. Park, J. E., Kim, B. B. & Park, E. D. Propane combustion over $Pt/Al_2O_3$ catalysts with different crystalline structures of alumina. *Korean J. Chem. Eng.* **32,** 2212-2219 (2015).
5. Hu, Q. et al. The precise decoration of Pt nanoparticles with Fe oxide by atomic layer deposition for the selective hydrogenation of cinnamaldehyde. *Appl. Catal. B,* **218,** 591-599 (2017).
6. Xu, D., Li, W., Duan, H., Ge, Q. & Xu, H. Reaction performance and characterization of $Co/Al_2O_3$ Fischer-Tropsch catalysts promoted with Pt, Pd and Ru. *Catal. Lett.* **102,** 229-235 (2005).
